# Argonaute bypasses cellular obstacles without hindrance during target search

**DOI:** 10.1038/s41467-019-12415-y

**Published:** 2019-09-26

**Authors:** Tao Ju Cui, Misha Klein, Jorrit W. Hegge, Stanley D. Chandradoss, John van der Oost, Martin Depken, Chirlmin Joo

**Affiliations:** 1 Kavli Institute of Nanoscience and Department of Bionanoscience, Delft University of Technology, Delft, The Netherlands; 2 Laboratory of Microbiology, Department of Agrotechnology and Food Sciences, Wageningen University, Wageningen, The Netherlands; 3 Present Address: Oxford NanoImaging, Oxford, UK

**Keywords:** Biophysical chemistry, Single-molecule biophysics, Small RNAs, Single-molecule biophysics

## Abstract

Argonaute (Ago) proteins are key players in both gene regulation (eukaryotes) and host defense (prokaryotes). Acting on single-stranded nucleic-acid substrates, Ago relies on base pairing between a small nucleic-acid guide and its complementary target sequences for specificity. To efficiently scan nucleic-acid chains for targets, Ago diffuses laterally along the substrate and must bypass secondary structures as well as protein barriers. Using single-molecule FRET in conjunction with kinetic modelling, we reveal that target scanning is mediated through loose protein-nucleic acid interactions, allowing Ago to slide short distances over secondary structures, as well as to bypass protein barriers via intersegmental transfer. Our combined single-molecule experiment and kinetic modelling approach may serve as a platform to dissect search processes and study the effect of sequence on search kinetics for other nucleic acid-guided proteins.

## Abstract (web summary)

Argonaute protein is loaded with small RNA and scans long stretch of sequences to find complementary target sites. Here, using single-molecule FRET and kinetic modelling, the authors showed that prokaryotic Argonaute protein binds target DNA loosely and slides along the DNA during target search.

## Introduction

Target recognition by oligonucleotide guides is essential in cellular development, differentiation, and immunity^1,2^. Argonaute (Ago) proteins are key mediators of the target interference process, utilizing short oligo-nucleotides (~20–30 nt) as guides for finding complementary target sequences^3,4^. The guide-target interaction initiates at the 5′ end of the guide, and progresses through Watson–Crick base pairing at the “seed” segment, which propagates along the guide, resulting in target interference upon completion^5^.

While eukaryotic Argonautes use RNA guides to target RNA, prokaryotic Agos (pAgo) have been demonstrated to use a variety of guides and targets^6–8^. Depending on the pAgo type, it uses either DNA or RNA guides to target single-stranded (ss) DNA, ssRNA or both^2^. The ability of pAgos to cleave ssDNA, but not double stranded DNA (dsDNA), suggests a physiological role as a host defense system against ss mobile genetic elements^6–8^. Recently, a new family of CRISPR-Cas systems that targets ssDNA—not dsDNA—have been discovered in archaea, suggesting that these defense systems may be more widespread than previously thought^9^.

The number of potential targets encoded in cellular DNA/RNA is vast^5,10,11^, and Ago needs to search long stretches of polymer before finding a canonical target. Single-molecule studies have shown that a mixture of excursions into solution and one-dimensional movements results in a search that is orders of magnitude more efficient than is possible without lateral diffusuion^12,13^. In a previous biophysical study we suggested that human Argonaute 2 (hAGO2) uses lateral diffusion along RNA for target search^14^. Yet, the degree of lateral diffusion remains unclear, as excessive usage of 1D diffusion would lead to redundant re-sampling of potential target sites and to problems at various roadblocks present on the target nucleic acids^15,16^.

In addition to complete dissociation into solution, intersegmental transfer, in which a protein transfers between two spatially close-by segments, has been shown to occur for DNA binding proteins, such as restriction enzyme EcoRV^17^. After binding to DNA non-specifically from solution, the protein diffusively scans only a limited section^13,18–20^, and dissociates into solution before rebinding to a new section. Use of such a mechanism would lead to reduced sampling redundancy, and the possibility to circumvent obstructions when proteins search for their targets.

Previous studies have shown that certain DNA/RNA-guided proteins interact with DNA through non-specific electrostatic interactions^21–23^, but the strength of these interactions and their behavior on roadblocks and secondary structures is not understood. Since these interactions are typically short-ranged^24–26^ and short-lived^14,21,23,26–29^, a method offering high spatiotemporal resolution is required to study these interactions. Here we make use of single molecule Förster Resonance Energy Transfer (FRET) to elucidate the mechanism of ssDNA target search by a mesophilic Ago from the bacterium *Clostridium butyricum* (*Cb*Ago). We show that *Cb*Ago does not remain in tight contact with the DNA backbone, enabling it to bypass secondary structures along the nucleic-acid chain—all while retaining the ability to recognize its target. After sliding locally, the protein is able to reach distant sites (>100 nt) along the DNA through intersegmental transfer and then resumes sliding. These different modes of facilitated diffusion allow Ago to rapidly search through nucleic acid segments, as well as to bypass substantial obstacles during target scanning.

## Results

### Single-molecule kinetics of CbAgo binding

**Fig. 1:**
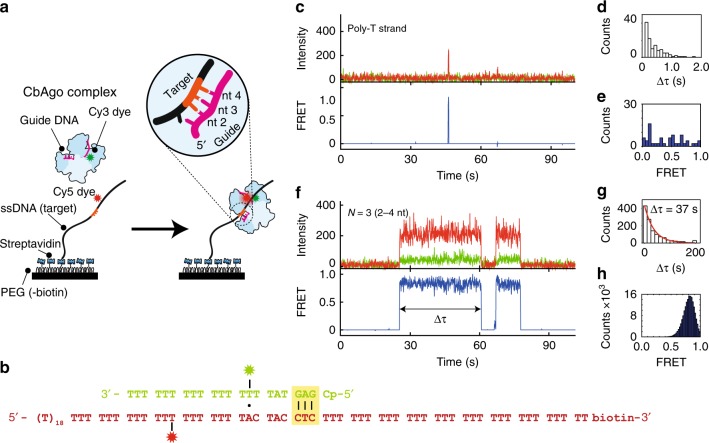
Single molecule imaging of target binding by siDNA:*Cb*Ago complex. **a** Immobilization scheme of the Argonaute-guide DNA complex. ssDNA is immobilized on a PEGylated quartz slide surface. Presence of the Ago-siDNA complex is detected by specific binding to target site (light yellow) resulting in high FRET. **b** Sequences of guide (green) and target DNA (red). Guide is labelled on the 9th nucleotide position from the 5′ side. **c** Representative FRET trace of a single molecule experiment at 100 mM NaCl showing a transient interaction between *Cb*Ago and a poly-T strand. Time resolution is 100 ms. **d** Dwell time distribution of the Argonaute in absence of target motif. **e** FRET values of the transient interactions of **d**. **f** Representative FRET trace of a single molecule experiment showing the interaction between *Cb*Ago and a 2–4 nt (*N* = 3) motif. **g** Dwell time distribution of *N* = 3 binding events with the mean dwell time of 37 s. **h** FRET histogram of binding events, showing a single FRET population for *N* = 3 at E = 0.78. Source data are provided as a Source Data file

To elucidate the complexity of the target search mechanism, we made use of the high spatial sensitivity of single-molecule FRET. We studied a minimal Argonaute complex that consists of *Cb*Ago, loaded with a 22-nt DNA guide (small interfering DNA, siDNA)^30^. By using total internal reflection fluorescence (TIRF) microscopy, we recorded the interactions of *Cb*Ago-siDNA with target DNA. Target DNA was immobilized on a PEG-coated quartz surface in a microfluidic chamber through biotin-streptavidin conjugation. Guide-loaded *Cb*Ago was introduced to the microfluidic chamber by flow. The target was embedded within a poly-thymine sequence and labelled with an acceptor dye (Cy5) (Fig. 1a). The guide construct was labelled at nt 9 from the 5′-end with a donor dye (Cy3) (Fig. 1b). A 532-nm laser excitation resulted in donor excitation when the protein loaded with the guide DNA interacted with the target DNA. Once the *Cb*Ago-siDNA complex became bound to the target, the proximity of the donor dye to the acceptor dye on the target resulted in high FRET efficiency. This was followed by a sudden disappearance of the signal, indicating that the complex dissociated from the target and diffused into the free solution. Freely diffusing molecules move too rapidly (~μs) in and out of the evanescent field for the current time resolution of the experimental setup (100 ms) and were therefore not recorded. We found that *Cb*Ago is not able to target dsDNA directly (Supplementary Fig. 1a–b). Likewise, when a ssDNA target with one base-pair complementarity to the seed motif of the guide was used, only transient interactions (~0.45 s) were detected (Fig. 1c, d), and no accurate binding profile could be extracted from the FRET histogram (Fig. 1e).

To observe target search that involves intrinsically transient interactions, we determined the optimal target motif for recording binding events. The optimal motif should provide binding events longer than our detection limit of 100 ms, but still lead to dissociation events within the time of our measurement (200 s). To determine the optimal motif, the complementarity between guide and target was incrementally extended from nt 2–8 of the guide, showing a gradually increasing dwell time of the Ago-siDNA complex. We found that increasing the number of complementary base pairs above six resulted in stable binding beyond the photobleaching time (Supplementary Fig. 1c). To maintain weak interactions, we continued our experiments using a siDNA with three-base complementarity (*N* = 3) with the target (nt 2–4) (Fig. 1f). This gives a well-defined FRET population in the FRET histogram (Fig. 1h), unlike one base-pair complementarity. Our estimation of the photobleaching rate (1.4 × 10^−3^ s^−1^) (Supplementary Fig. 1d) was an order of magnitude lower than the dissociation rate (2.7 × 10^−2^ s^−1^) (Fig. 1g), indicating that photobleaching does not affect our estimation of the dissociation rate.

### Lateral diffusion of *Cb*Ago

It was previously shown that an Ago-guide complex does not directly bind a specific target site from solution, but rather binds non-specifically to random positions along a surfaced-immobilized nucleic acid construct^14^. Such non-specific interactions of *Cb*Ago-siDNA along target DNA are too short-lived to resolve in the absence of a canonical target motif (Fig. 1c), and in the presence of such a motif there was still no lateral diffusion visible (Fig. 1f). As we were unable to resolve lateral diffusion by *Cb*Ago from non-specifically bound regions to the target, we questioned whether the observed stable signal for three complementary base pairs is due to stable binding to the target or contains lateral excursions away from the target but below our time resolution. In case of the latter, measured apparent dwell times (Fig. 1g) would consist of the combined dwell times of many target escapes through lateral diffusion, each followed by rapid recapture below the detection limit, before *Cb*Ago eventually unbinds from the DNA (Supplementary Fig. 1g). We show that such a process of repeated recapture would result in an exponential distribution of apparent dwell times, in accordance with Fig. 1g (see Supplementary Note 1).

**Fig. 2:**
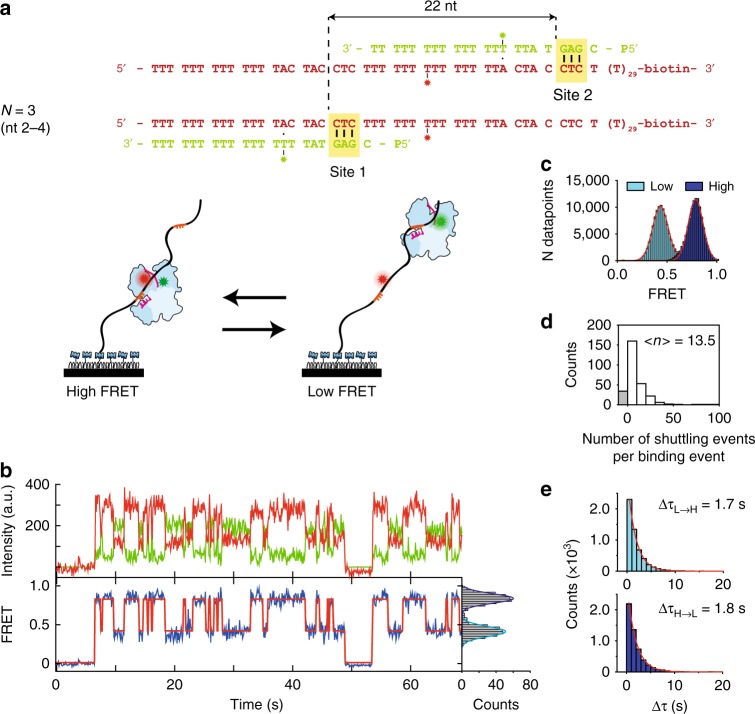
Shuttling signature of *Cb*Ago appears in presence of two targets. **a** In the top, the DNA sequence of guide and target for 22 nt separation between targets. Here the distance is defined as the distance from beginning of a target to the beginning of the next target. The placement of the second target (site 2) results in the appearance of an additional FRET signal, with lower FRET efficiency. **b** (Top) Representative shuttling trace of a 22 nt separation tandem target at 100 mM NaCl for *N* = 3. (Bottom) The corresponding FRET states (blue) with the fitted HMM trace on top (red). (Right) FRET histogram of the respective time trace. Time resolution is 100 ms. **c** FRET histograms of respective states, with peaks at 0.43 and 0.78. **d** Shuttling event distribution for the same conditions (*n* = 309). Bin size = 10. On average 13.5 shuttling events take place before dissociation. The gray bar (*n* = 33) marks binding events followed by dissociation (no shuttling). **e** Dwell time distributions of the transitions from low FRET state to high FRET state (top) and vice versa (bottom). Source data are provided as a Source Data file

To overcome the temporal resolution limit, we adopted a tandem target assay^14,31^. While lateral diffusive excursions from a trap are too short-lived to be resolved in the presence of only a single target, a second target can trap an excursion for long enough to be observed. We placed two identical optimal targets (site 1 and site 2) separated by 22 nt (Fig. 2a) along the DNA construct. Both targets base pair only with the first three nucleotides (nt 2–4) of the guide bound by *Cb*Ago. As the second target is located further away from the acceptor dye, binding the second target results in a lower FRET efficiency than binding the first target. This difference in FRET values allows us to determine which of the two targets *Cb*Ago-siDNA is bound to (Fig. 2b). The respective distance and FRET efficiency between the first binding site (site 1) and the acceptor dye (Cy5) remained the same as for the single target assay (E~0.78), while an additional peak appeared at a lower FRET efficiency for the second target (E~0.43, Fig. 2c). After binding to one of the target sites, a majority of the binding events (87.8%) resulted in *Cb*Ago-siDNA shuttling to the other target without loss of FRET signal. Under our standard experimental condition (100 mM NaCl), an average of 13.5 shuttling events occur per binding event (Fig. 2d). When the experiment was repeated for guides and targets with complementarity increased to *N* = 6 (nt 2–7), only 15.1% of the traces showed the shuttling signature within our time window (Supplementary Fig. 2f). This shows that the shuttling signature is controlled by interactions between *Cb*Ago-ssDNA and the target motif. With a 6-nt match, the target is strongly bound, and we are less likely to observe a shuttling event within our observation window.

Interestingly, the average dwell time of the first target (Fig. 1g) decreased from 37 s to 1.7 and 1.8 s after adding a second target in its vicinity (Fig. 2e). This observation is in agreement with our lateral diffusion model, since with close-by targets, each sub-resolution diffusive excursion has some probability to be caught at the opposing target. To further test our claim that the transition between targets occur through lateral diffusion, we use single-molecule analysis software^32^ to extract the average time between shuttling events (Δ*τ*_shuttle_) from traces (Supplementary Fig. 3).

### Kinetic modelling of lateral diffusion

To determine how lateral diffusion contributes to the shuttling, we kinetically model how Δ*τ*_shuttle_ depends on the distance between traps. The DNA construct is modelled as a series of binding sites along which *Cb*Ago will perform an unbiased random walk by stepping to neighboring nucleotides. The rate of stepping away from the target is *k*_esc_ in both directions, while at non-specific sites (poly-T), stepping is assumed to be near instantaneous—an approximation justified by the fact that lateral excursions are never resolved in the experiments. The time needed for FRET transitions to occur (named “shuttling time”, Δ*τ*_shuttle_) is equivalent to the apparent dwell time at a single FRET state. In Supplementary Note 2 we construct a diffusive model capturing the effect of Ago’s repeated retrapping before shuttling to the other trap. The model shows that the shuttling time from the target grows linearly with the separation *x*_target_ between the targets

1 $$\Delta \tau _{{\mathrm{shuttle}}}\left( {x_{{\mathrm{target}}}} \right) = \frac{{x_{{\mathrm{target}}}}}{{k_{{\mathrm{esc}}}}}$$

**Fig. 3:**
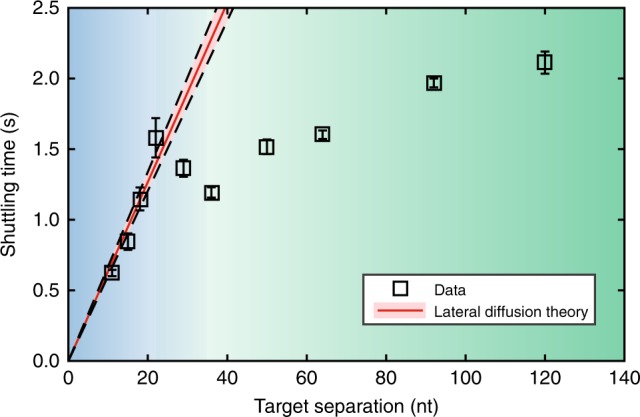
*Cb*Ago shuttling behaviour differs across short and large distances. Shuttling time is plotted vs. distance between targets. Squares indicate the mean shuttling time for each DNA construct. The plotted error bars indicate the 95% confidence interval of 10^5^ bootstrapped dwell times. The red line indicates the lateral diffusion model where the first four datapoints are fitted with *k*_esc_ = 15.8 s^−1^. The shaded red region indicates its 95% confidence interval derived from bootstrapping the data. The blue region indicates where the shuttling time follows lateral diffusion theory. This theory breaks down for larger distances (green). Source data are provided as a Source Data file

The linear dependence of the shuttling time with trap separation might seem puzzling at first, given that diffusive timescales usually show a quadratic dependence on distances. Here though, it is not the diffusive steps themselves that directly contributes to the shuttling time, but rather the changing probability to getting retrapped before shuttling. In support of this model, we observed that the apparent shuttling time Δ*τ*_shuttle_ (*x*_target_) increases approximately linearly when the distance between the targets increases through 11, 15, 18, and 22 nt (Fig. 3). A fit to Eq. 1 reveals that *Cb*Ago-siDNA complexes escape the target site at a rate of 15.8 times per second (*k*_esc_ = 15.8 s^−1^) in either direction.

### Ago probes for targets during lateral diffusion

**Fig. 4:**
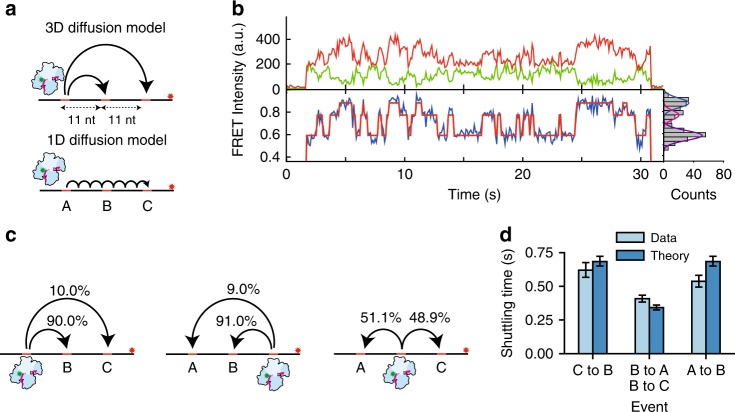
*Cb*Ago undergoes short range diffusion through correlated steps. **a** Models for target translocation at short range. In the 3D diffusion model, target dissociation occurs from A followed by random 3D diffusion through solution. In effect, the neighboring two targets (B and C) will compete for binding. In the lateral diffusion model, the *Cb*Ago complex will have to bypass the adjacent target B before binding to target C. **b** Representative FRET trace showing the shuttling behavior between three targets. Top: donor (green) and acceptor (red) intensities. Bottom: FRET trace (blue) and HMM assigned states (red). Right: The fitted states from this data trace with dark blue: state C, pink: state B and purple state A. **c** Transition probabilities from state A to B,C (left), from state C to A and B (middle) and from state B to A or C (right). **d** Experimental values of the shuttling time of the three target construct were compared against the parameter-free theoretical model that only uses the *k*_esc_ = 15.8 s^−1^ from Fig. 3. Error bars indicate the 95% confidence interval acquired from 10^5^ bootstraps (see Supplementary Information). Source data are provided as a Source Data file

Next, we placed a third target on the tandem construct (Fig. 4a), keeping the distance between each set of neighboring targets well within the regime for which we find good agreement to Eq. 1 using the assay discussed above (i.e. at 11 nt trap separation, see Fig. 3). We observed three different FRET levels, corresponding to *Cb*Ago getting trapped at the three different targets (Fig. 4b, Supplementary Fig. 4a). Using Hidden Markov Modelling (HMM), states can be assigned (Fig. 4b) and transition probabilities can be extracted (Fig. 4c). If *Cb*Ago returns to solution between binding targets, transitions between any pair of targets will be equally probable, resulting in equal effective rates between all targets. However, if lateral diffusion dominates, transitions between adjacent sites will be favored. The transition probabilities (Fig. 4c) indicate that over 90% of the transitions between the two outer targets (from state A to C, or from C to A) proceed through the intermediate target site (state B). The rate to transfer from B to C and B to A is twice as much as that of the opposite path (A to B or C to B). Using the fitted escape rate from above, *k*_esc_ = 15.8 s^−1^, we predict similar shuttling times based on our theoretical model for lateral diffusion (Fig. 4d, Supplementary Notes 3–4). With no more free-parameters remaining for this prediction, we take this experimental agreement with our prediction as further evidence of lateral diffusion.

It is noteworthy that there are about 10% direct transitions from A to C and C to A without any intervening dissociation. The exponential distribution of the dwell times (Supplementary Fig. 4b) suggests that at our current time resolution this 10% may be either due to missed events or due to the existence of an additional translocation mode through which Ago is able to bypass the intermediate target.

### Ago target search is unhindered by junctions and proteins

**Fig. 5:**
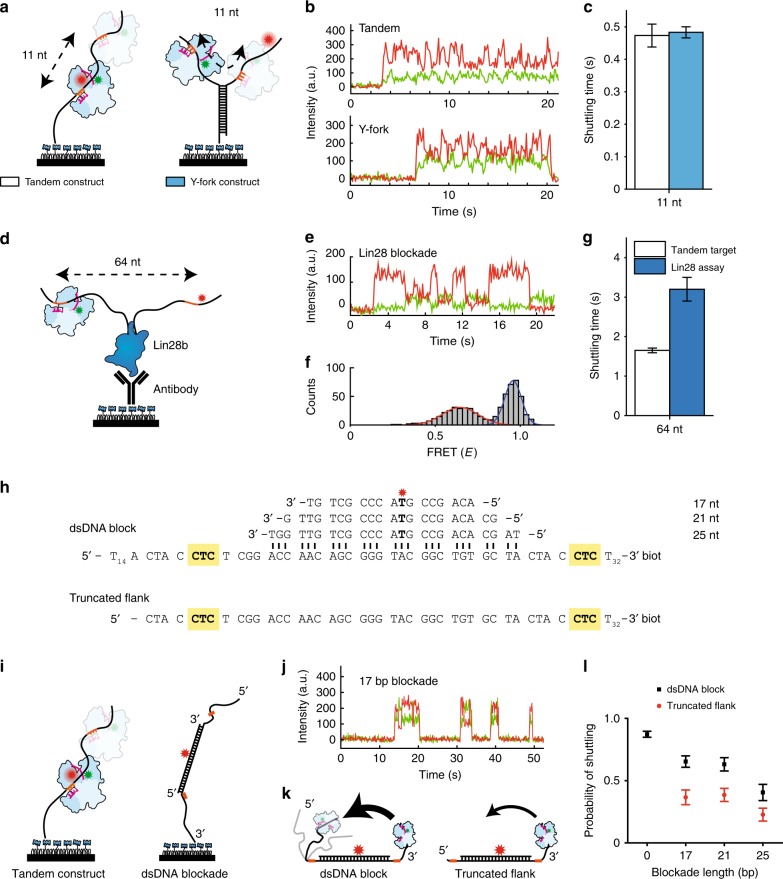
Argonaute can overcome structural and protein barriers. **a** Schematic drawing of the tandem target assay (left) and the Y-fork assay (right) with 11 nt separation between targets. **b** Representative shuttling traces of the tandem target assay (top) and Y-fork assay. **c**, The shuttling time of the Y-fork junction (blue bar) compared with the tandem assay (white bar). The experimental data of both sets were taken on the same days. Error bars indicate the 95% confidence interval acquired from 10^5^ bootstraps **d**, Schematic drawing of the His-Lin28b blockade assay, where targets are separated by 64 nt. Immobilization happens through a biotin-anti-His antibody. **e** Example of a shuttling trace with Lin28b located between two targets. Exposure time is 100 ms. **f** FRET histogram (molecules *n* = 46) fit with two Gaussian functions (*E* = 0.64 for red fit and *E* = 0.95 for dark blue fit). **g** The shuttling time of the Lin28 assay compared with the tandem target assay for 64 nt separation between targets. **h** Sequences used for the dsDNA block assay, indicating the base pairing between a 17, 21, and a 25 nt long blockade and the target strand. The dsDNA block construct has a 19 nt flank on the 5′ side, whereas the “truncated flank” has a 4 nt flank. **i** Schematic of a dsDNA block assay, where the CTC targets are highlighted with orange. **j** Representative trace of binding and shuttling of CbAgo on a 17 bp blockade DNA construct. **k** (left) Schematic of dsDNA block construct with full length flanks. (right) schematic of the truncated version where the flank on the 5′ side is removed. The thickness of the arrows indicate the observed shuttling probability. **l** The probability of shuttling upon binding to a CTC target plotted vs. the blockade length (none, 17, 21, and 25 nt) for full length flanks (black squares) and for the truncated flanks (red circles). Error bars are given by the 95% confidence interval acquired from 10^5^ bootstraps. Source data are provided as a Source Data file

Secondary structures are commonly found in mRNA and are also predicted to exist in single stranded viruses^33,34^. It is not known whether *Cb*Ago is able to bypass the numerous junctions it encounters upon scanning a DNA segment. To examine this, a Y-fork structure (DNA junction) was introduced as a road block between two targets (Fig. 5a), while keeping their separation (11 nt) the same as in the tandem target variant (Supplementary Fig. 4f). The construct was designed such that the labelled target was partially annealed at the stem with a biotinylated target, thus only annealed constructs were observable on the surface of the microfluidic device. When *Cb*Ago binds to either of the two targets, it can reach the other target only by crossing the junction. Our measurement showed that there was no significant difference in shuttling time between the standard tandem-target construct and the Y-fork construct (Fig. 5b, c), indicating that the Y-fork does not impede any of the lateral diffusion modes present. We have previously observed that the *Cb*Ago-siDNA complex is not able to stably bind to dsDNA^30^, demonstrating that the protein cannot simply track the backbone of dsDNA (Supplementary Fig. 1a–b). Thus, our result suggests that the Ago-siDNA complex does not maintain tight contact with DNA during lateral diffusion. Maintaining a weak interaction with the DNA molecule allows *Cb*Ago-siDNA to move past the junction.

Next, we questioned whether *Cb*Ago is also able to overcome larger barriers, such as proteins which cannot reasonably be traversable through sliding alone. Lin28, a sequence-specific inhibitor of let-7 miRNA biogenesis, has been found to associate sequence specifically to RNA and DNA^35^. His-tagged Lin28 was immobilized on the surface of the microfluidic chamber (Supplementary Fig. 4d) after which a fluorescent ssDNA fragment was added containing a central Lin28 binding motif and an Ago target motif on either side (Fig. 5d, Supplementary Fig. 4g). The presence of the protein blockade did not preclude Ago from reaching the distal site (Fig. 5e) but noticeably broadened the FRET peak (Fig. 5f), possibly due to protein-protein interactions. Although the shuttling rate was lowered from 0.60 s^−1^ to 0.27 s^−1^ (Fig. 5g, Supplementary Fig. 4e), Ago is able to bypass the obstacle. Since short-range lateral movement is now blocked by the protein barrier, Ago’s ability to move between targets demonstrates that the target search process also allows for intersegmental transfer, in accordance with our observation that the middle target is sometimes skipped when transitioning between the outer targets in Fig. 4c.

### Flexibility of DNA segments allows Ago to bypass blockades

Since Ago was observed to be able to bypass junctions and proteins, we questioned whether Ago could bypass other large-profile barriers. Previously, we observed that Ago only interacts transiently with dsDNA (Supplementary Fig. 1a–b) and thus we repurposed dsDNA as an extended blockade. We made a construct analogous to the tandem target construct used in Fig. 2a, but the targets were separated by 36 nt and complementary strands of 17, 21, and 25 nt were annealed to the region in between the targets (Fig. 5h, i).

For the construct with a 17-nt blockade we observed a large number of shuttling events (shuttling probability 65.3% upon binding) indicating that a dsDNA blockade does not prohibit CbAgo from reaching the other site (Fig. 5j and Fig. 5l black squares). Upon extending the length of the dsDNA blockade, to 21 nt and 25 nt, we noticed a drop in the percentage of shuttling events (63.1% and 40.4% respectively) although shuttling still persisted (Supplementary Fig 5). Since the stiff segment of dsDNA decreases the shuttling probability, we conclude that Ago relies on the flexibility of segments for lateral diffusion.

To further investigate the contribution of DNA flexibility, we used another construct which was shortened (by 15 nt from 19 nt) from the 5′ side (Fig. 5h bottom sequence). Here, ssDNA coiling was no longer possible from the 5′ side of the DNA construct (Fig. 5k). We measured a significant decrease (~50%) in shuttling probability for all three blockades compared to the untruncated construct (Fig. 5l), which supports that Ago relies on the flexibility of DNA segments when transferring between them.

### Ago uses hops to access distant DNA segments

Sliding is not expected to dominate across large distances, as the linear increase in shuttling time (Eq. 1) would render the search process prohibitively slow. However, when *Cb*Ago was studied with tandem targets that were separated 36 nt or more, we observed that the shuttling still persisted across larger distances (Fig. 3, green region, Supplementary Table 1 and Supplementary Fig. 6). Together with the evidence of intersegmental transfer above, and the fact that the ssDNA can easily be coiled back to bring the second target close to the Ago protein^36^, we speculate that there is a second mechanism of lateral diffusion**:** after local scanning for the target through sliding, the *Cb*Ago complex hops to a different part of the DNA that has looped back into proximity of the complex. From this point on, we refer to these hops as intersegmental transfer in accordance with the current literature^17,37^ (Supplementary Fig. 7). This intersegmental transfer mechanism would enable *Cb*Ago to travel to new sites without fully dissociating, and rescanning of the same sections would be minimized^16,19^.

Based on the dependence of the single-target off-rate on the ionic strength (Supplementary Fig. 1f), we expect the rate of the intersegmental transfer to also be dependent on salt concentration, while sliding should only be moderately effected since it has no net effect on the ion condensation along the substrate. In order to test the hypothesis that short-ranged lateral diffusion is governed by sliding and long-range diffusion is governed by intersegmental transfer, we altered the ionic strength of the buffer solution from 10 mM NaCl to 200 mM NaCl. Here, we expect the degree of DNA coiling not to be significantly affected by the change in salt concentration, since the persistence length is only expected to vary between ~20 Å and 14 Å when exchanging the buffers, and in both buffers it is smaller than the contour length of the constructs^36^.

**Fig. 6:**
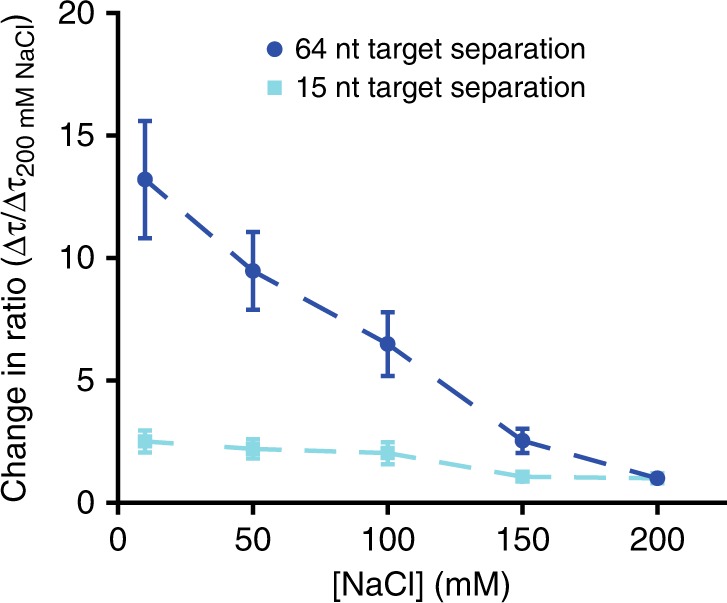
Argonaute target search is characterized by sliding  and intersegmental transfer. The relative change in shuttling time of two constructs from Fig. 3, 64 nt separation (dark blue circles) and 15 nt separation (light blue squares), normalized against Δ*τ*_shuttle_ at 200 mM NaCl. Errors of the ratio were determined through bootstrapping 10^5^ times the ratio of Δ*τ*_shuttle_/${\mathrm{\Delta }}\tau _{{\mathrm{shuttle}}}^{200\,{\mathrm{mM}}\,{\mathrm{NaCl}}}$. Source data are provided as a Source Data file

We used dual-target constructs with 15-nt separation and 64-nt separation (Fig. 6), taken from the two different regions in Fig. 3 (indicated by blue and green shading). At a separation of 64 nt, we observed a 13-fold increase of the shuttling rate when increasing the salt concentration from 10 mM NaCl to 200 mM NaCl. In contrast, we observed that for the dual-target construct with 15-nt separation, the shuttling time changed roughly only two-fold for the same change in ionic strength (Fig. 6)—a modest change compared to 13-fold of the dual-target constructs with 64-nt separation. We take the relative ionic-strength insensitivity of shuttling times for 15-nt trap separation as evidence of translocation being dominated by sliding over short distances. In contrast, given the relative ionic-strength sensitivity for the 64-nt construct, the Ago complex is here unlikely to first reach the distal site through sliding only, and requires partial dissociation from the DNA strand.

In conclusion, lateral diffusion during *Cb*Ago target search is governed by two distinct modes. For short distances, lateral diffusion takes place through a sliding process characterized by loose contact with the DNA strand. This allows the protein to pass over secondary structures as if it glides. To traverse larger distances, *Cb*Ago is able to take advantage of the fact that the softness of the substrate allows it to bend back on itself to enable frequent intersegmental transfer between nearby segments (Supplementary Fig. 8).

## Discussion

Within a vast number of potential targets, Ago-guide complexes have to minimize the time spent unproductively diffusing through solution or redundantly checking off-targets, as timely regulation is crucial for both cell development and host defense^38^. Our single-molecule study shows that Argonaute from *C. butryicum* (*Cb*Ago) uses a loose sliding mode to bypass junctions and relies on intersegmental transfer to cover larger distances and to bypass substantial barriers.

We have shown that bacterial Ago binds DNA loosely and slides along the DNA to locally scan for complementary targets. While such sliding mechanism has been characterized for several proteins^13,18,39,40^, little was previously known for DNA/RNA-guided target searchers like Ago. Proteins searching along nucleic acids with secondary structures may be blocked from sliding further. However, this does not seem to be true for Ago. Instead, the loose interaction with the substrate allows the protein to slide past junctions while still probing potential target sequence through base pairing. To the best of our knowledge, the ability to bypass junctions and roadblocks along single-stranded DNA using loose-contact sliding has not been reported for any nucleic-acid-guided proteins. In addition, we show that the loose binding further allows Ago to move to a new segment via intersegmental transfer, reducing redundant scanning of the same segment and allowing Ago to bypass large-profile roadblocks.

The ability of *Cb*Ago to target specifically ssDNA but not dsDNA^30^ (Supplementary Fig. 1a–b) suggests a role as host defense against mobile genetic elements and ssDNA viruses. In environments where ssDNA viruses can be abundant, such as in sea water, fresh water, sediment, terrestrial, extreme, metazoan-associated and marine microbial mats^41–43^, pAgo’s targeting ssDNA would be very beneficial for the host. Upon entry in the infected cell, ssDNA binding and recombination proteins may associate with the invading nucleic acid, and DNA polymerase will start to generate the second strand. In addition, it is anticipated that secondary structures will be formed in the ssDNA viral genome^33^. This will generate road blocks that may affect scanning by defense systems such as restriction enzymes but—as shown here—not Argonaute. Likewise, insertion of transposons in prokaryotes often proceeds via a ssDNA-intermediate state^44–46^, and pAgos may here encounter the same type of obstacles. In case of ssRNA, both in prokaryotes and in eukaryotes, it is well known that complex secondary structures can be formed by base pairing different anti-parallel RNA segments^47–50^. The presence of secondary structures suggests that it is necessary for Agos to glide—the type of loosely bound sliding we report—past such roadblocks to enable search along ssRNA. Based on the functional and structural similarities of prokaryotic Agos and eukaryotic Agos^2,14^, we expect eAgo to also slide past RNA secondary structures, minimizing time spent trapped at such structures.

The effect of lateral diffusion on the total target search time is dependent on the roughness of the energy landscape that the DNA binding protein encounters once it binds non-specifically. We have shown how to determine the escape time for a 3-nt complementary target. This can be extended to estimate the escape time for any complementarity and consequently the diffusion constant on DNA with any base composition^51^. Here we have inferred a 15.8 s^−1^ escape rate from the 3-nt CTC guide sequence (Fig. 3), indicating that if a target strand were to consists only of GA in repeating order, the effective diffusion coefficient $D = \frac{{dx^2}}{{2dt}} = \frac{{nt^2}}{{2\left( {2 \ast k_{esc}} \right)^{ - 1}}} = {\mathrm{nt}}^2k_{esc} = 15.8\frac{{{\mathrm{nt}}^2}}{s}$. Changing the number of base-paring nucleotides as well as the identity of nucleotides in the guide/target could provide insights into how sequence variation would affect the rate of diffusion for other nucleic acid proteins.

Since the guide strand only provides the specificity needed for accurate targeting, lateral diffusion could be reliant on the non-specific surface interactions with the protein. We envision that the positive surface charge distribution inside the Ago cleft could orient Ago with the guide toward the negatively charged nucleic acid strand (Supplementary Fig. 9), thereby promoting target interrogation while traveling along the target strand. It is unknown whether Ago is able to scan each base during this process or whether it skips over nucleotides. For our triple-target construct, we have observed that 90% of the time the middle target traps Ago. It will be of interest to investigate whether this level of effective target trapping is achieved by a low trapping efficiency offset by repeated passes over the target.

For a longer range target search, we have observed that at distances >100 nt separation, the shuttling time remains well below what would be expected for sliding (Fig. 3). We show that coiling of the ssDNA (persistence length ~1 nm) may bring distant segments in close proximity, allowing intersegmental transfer over longer distances (beyond ~30 nt target separation), and so speeding up lateral diffusion. Interestingly, Ago cannot use intersegmental transfer to cover shorter distances, as implied by the sudden increase in shuttling time when the trap separation goes below 30 nt (Fig. 3). Experimentally, one could further investigate the nature of intersegmental transfer through a combined tweezer-fluorescence single-molecule assay, where forces strong enough to pull on entropically coiled ssDNA can be applied^17,37^. Furthermore, theoretical modelling and additional experiments are required in order to establish to what extent partitioning the search modes on different length scales will allow nucleic-acid-guided proteins to optimize the search process^52–55^ since the absence of cooperative binding was recently reported for another Ago system^29^.

We hypothesize that similar target search strategies may be used by Agos from different families, which are structurally and functionally similar^2^. For example, in RNA induced transcriptional silencing (RITS), guide-loaded AGO1 binds to a transcript after which other proteins are recruited for heterochromatin assembly^56,57^. Similarly, in the piRNA pathway PIWI proteins associate with piRNA in germline cells to bind and cleave transposon transcripts in the cytoplasm^58–60^ or to nascent RNA in the nucleus in order to induce heterochromatin formation^61^. In each of these functions, the reliance on guide-complementary for sequential target search likely necessitates the usage of facilitated diffusion strategies to optimize the search time for proper regulation of cell development or gene stability.

## Methods

### Purification of CbAgo

The CbAgo gene was codon harmonized for E.coli Bl21 (DE3) and inserted into a pET-His6 MBP TEV cloning vector (Addgene plasmid # 29656) using ligation independent cloning. The CbAgo protein was expressed in E.coli Bl21(DE3) Rosetta™ 2 (Novagen). Cultures were grown at 37 °C in LB medium containing 50 µg ml^−1^ kanamycin and 34 µg ml^−1^ chloramphenicol till an OD600nm of 0.7 was reached. CbAgo expression was induced by addition of isopropyl β-d-1-thiogalactopyranoside (IPTG) to a final concentration of 0.1 mM. During the expression cells were incubated at 18 °C for 16 h with continues shaking. Cells were harvested by centrifugation and lysed, through sonication (Bandelin, Sonopuls. 30% power, 1 s on/2 s off for 5 min) in lysis buffer containing 20 mM Tris-HCl pH 7.5, 250 mM NaCl, 5 mM imidazole, supplemented with a EDTA free protease inhibitor cocktail tablet (Roche). The soluble fraction of the lysate was loaded on a nickel column (HisTrap Hp, GE healthcare). The column was extensively washed with wash buffer containing 20 mM Tris-HCl pH 7.5, 250 mM NaCl and 30 mM imidazole. Bound protein was eluted by increasing the concentration of imidazole in the wash buffer to 250 mM. The eluted protein was dialysed at 4 °C overnight against 20 mM HEPES pH 7.5, 250 mM KCl, and 1 mM dithiothreitol (DTT) in the presence of 1 mg TEV protease (expressed and purified according to Tropea et al.^62^) to cleave of the His6-MBP tag. Next the cleaved protein was diluted in 20 mM HEPES pH 7.5 to lower the final salt concentration to 125 mM KCl. The diluted protein was applied to a heparin column (HiTrap Heparin HP, GE Healthcare), washed with 20 mM HEPES pH 7.5, 125 mM KCl and eluted with a linear gradient of 0.125–2 M KCl. Next, the eluted protein was loaded onto a size exclusion column (Superdex 200 16/600 column, GE Healthcare) and eluted with 20 mM HEPES pH 7.5, 500 mM KCl and 1 mM DTT. Purified CbAgo protein was diluted in size exclusion buffer to a final concentration of 5 μM. Aliquots were flash frozen in liquid nitrogen and stored at −80 °C.

### Purification of His-tagged Lin28b

The protein was prepared following the protocol of Yeom et al.^63^. Briefly, recombinant Lin28b was prepared by subcloning cDNA with BamHI and XhoI into pET28-a vector (Novagen). Subsequently, the strain was transformed to *E. coli* BL21-RIL strain. The expression and purification of recombinant Lin28b was performed according to the manufacturer’s protocol.

### Single molecule experimental setup

Single molecule FRET experiments were performed with an inverted microscope (IX73, Olympus) with prism-based total internal reflection. Excitation of the donor dye Cy3 is done by illuminating with a 532 nm diode laser (Compass 215 M/50 mW, Coherent). A 60X water immersion objective (UPLSAPO60XW, Olympus) was used for collection of photons from the Cy3 and Cy5 dyes on the surface, after which a 532 nm long pass filter (LDP01-532RU-25, Semrock) blocks the excitation light. A dichroic mirror (635 dcxr, Chroma) separates the fluorescence signal which is then projected onto an EM-CCD camera (iXon Ultra, DU-897U-CS0-#BV, Andor Technology). All experiments were performed at an exposure time of 0.1 s at room temperature (22 ± 0.1 °C).

### Fluorescent dye labeling of nucleic acid constructs

All DNA constructs were ordered from ELLA Biotech. Nucleic acid constructs that have an internal amino modification were labeled with fluorescent dyes based on the CSHL protocol^64^. One microliter of 1 mM of DNA/RNA dissolved in MilliQ H20 is added to 5 μL labeling buffer of (freshly prepared) sodiumbicarbonate (84 mg/10 mL, pH 8.5). One microliter of 20 mM dye (1 mg in 56 μL DMSO) is added and incubated overnight at 4 °C in the dark, followed by washing and ethanol precipitation. Concentration of nucleic acid and labeling efficiency was determined with a Nanodrop spectrophotometer.

### Single molecule chamber preparation

Quartz slides were coated with a polyethylene-glycol through the use of amino-silane chemistry. This is followed by assembly of microfluidic chambers with the use of double sided scotch tape. For a detailed protocol, we refer to^65^. Further improvement of surface quality occurs through 15 min incubation of T50 and 5% Tween20^66^ after which the channel is rinsed with 100 μL T50 buffer. Streptavidin (5 mg/mL) was diluted in T50 to 0.1 mg/mL. Fifty microliter of this solution is then flowed inside the chamber. This is followed by incubation for 1 min followed by rinsing with approximately 10-fold the volume of the chamber with T50 (10 mM Tris-HCl [pH 8.0], 50 mM NaCl). In all, 100 pM of DNA/RNA target with biotin construct is then flushed in the chamber, followed by 1 min incubation. This is followed subsequently by rinsing with T50. The chamber is subsequently flushed with CbAgo buffer, containing 50 mM Tris-HCl [pH 8.0], 1 mM Trolox, 1 mM MnCl2, 100 mM NaCl. Guide-loading of apo-CbAGO occurs by incubation of the protein (10 nM) with 1 nM guide construct in a buffer containing 50 mM Tris-HCl [pH 8.0], 1 mM Trolox, 1 mM MnCl2, 100 mM NaCl, 0.8% glucose at 37 °C for 30 min. Following incubation, glucose oxidase and catalase is added (0.1 mg/mL glucose oxidase) after which the sample is flushed in the microfluidic chamber containing the DNA targets.

### Lin28 assay

Immobilization of Lin28b occurred in the following way: 50 μl of streptavidin (0.1 mg/mL) in T50 is flowed inside the chamber and incubated for 1 min. After this, the chamber is rinsed with approximately 100 μL of T50. One microliter of Anti-6X His tag® antibody (Biotin) diluted 100-fold in T50 and subsequently flowed inside the chamber. After 5 min, the chamber is rinsed with 100 μL of T50. Stock of Lin28b (100 μM) is diluted to 100 nM and incubated with the target DNA (10 nM) and 10 mM MgCl_2_ for 5 min, after which the solution is flushed inside the chamber, followed by incubation of 5 min. Lastly, the CbAgo buffer is flushed inside the chamber. Guide-loading of apo-CbAgo occurs in the same way as described above (Single molecule chamber preparation) after which the CbAgo:siDNA complex is also flushed inside the chamber.

### Data acquisition and analysis

Fluorescence signals are collected at 0.1-s exposure time unless otherwise specified. For 7-nt target separation, 30-ms exposure time is used. Time traces were subsequently extracted through IDL software using a custom script. Prior to data collection, the location of targets (Cy5 labeled) are found by illuminating the sample with the 637 nm laser. Through a mapping file, it subsequently collects the individual intensity hotspots in both the donor and acceptor channel and pairs them up through the mapping file, after which the traces are extracted. During the acquisition of the movie, the green laser is used. Only at the end, the red laser is turned on once more to check for photobleaching of the red dye. Traces containing the fluorescence intensity from the donor and acceptor signal are manually pre-selected occurs through the use of MATLAB (Mathworks), disregarding artefacts caused by non-specific binding, additional binding to neighboring regions and photobleaching.

### Determination of dissociation rate

Binding of Argonaute complex to a single target results in a sudden increase of acceptor signal. The length of these interactions was quantified through a custom script in MATLAB 2015b based on a thresholding algorithm. Briefly, a histogram was made of every data trace, from which the lowest population was fitted with a Gaussian peak. The resulting mean value and standard deviation are then used to distinguish binding events. Intensities that exceeded five times the standard deviation of the baseline (noise) were recognized as a potential binding event. Events that were recognized as potential binding events were marked by the script with a marker for individual checking. Subsequently, the duration of these events were collected and plotted in Origin. Some interactions (at low ionic strength) (Supplementary Fig. 1f) were beyond the observation window of our setup. Hence only a lower limit of the dissociation time could be given. The collected dwell times were bootstrapped through custom code using standard bootstrap algorithms provided by MATLAB. From the resulting distribution, the 95% percentile confidence interval is taken as the error.

### HMM analysis

For assigning states to the FRET traces, a HMM software package is used from Van et al.^32^, which can be found on their github repository (https://ebfret.github.io/**)**. Their software package is optimized for immobilized donor dye molecules on the surface. Here, we immobilize the acceptor dye molecule and hence when no molecule is present, the zero intensity signal in both channels results in large variations in FRET signal, which will result in false positives for the ebFRET software.

Increasing the donor signal and hence artificially creating an extra stable “zero FRET state” is adequate for our purposes, as the distinction between bound and unbound molecules is still made. For the analysis of shuttling traces from constructs where the subseed targets are located far away, the low FRET bound state becomes almost indistinguishable from donor only. Here, this method proves adequate in separating the two states (Supplementary Fig. 3).

After assigning states to the collected data, the dwell times for low FRET → high FRET and vice versa are extracted. The experimental data shows that there is only one rate-limiting step, in accordance with our theoretical analysis shown below. Using maximum likelihood estimation, the lifetime Δ*τ*_shuttle_ of the single-exponential distribution $P\left( t \right) = \tau _{{\mathrm{shuttle}}}^{ - 1}e^{ - t/{\mathrm{\Delta }}\tau _{{\mathrm{shuttle}}}}$ was extracted (the empirical average dwell time equals the ML estimator of Δ*τ*_shuttle_). The 95% confidence interval was extracted using empirical bootstrapping.

### Reporting summary

Further information on research design is available in the Nature Research Reporting Summary linked to this article.

## Supplementary information

Supplementary Information

Peer Review File

Reporting Summary

Source Data

## Data Availability

A reporting summary for this Article is available as a Supplementary Information file. The source data underlying Figs. 1–6 and Supplementary Figs. 1–4 are provided as a Source Data file. All data is available from the corresponding author upon reasonable request.

## Code availability

The code that was used to analyze the data is available from the corresponding authors upon request.
