# Supplementary material for: Argonaute bypasses cellular obstacles without hindrance during target search
Source: Nat Commun. 2019 Sep 26;10:4390. doi: 10.1038/s41467-019-12415-y (PMC6763497; doi:10.1038/s41467-019-12415-y)
Supplement: Supplementary file 1 — Supplementary Information [file 41467_2019_12415_MOESM1_ESM.pdf]

Supplementary Information

Argonaute bypasses cellular obstacles without hindrance during target search

Tao Ju Cui[1], Misha Klein[1], Jorrit W. Hegge[2], Stanley D. Chandradoss[1, 3], John van der Oost[2] Martin Depken[1, *], Chirlmin Joo[1, *]

[1] Kavli Institute of Nanoscience and Department of Bionanoscience, Delft University of Technology, Delft, The Netherlands

[2] Laboratory of Microbiology, Department of Agrotechnology and Food Sciences, Wageningen University, Wageningen, The Netherlands

[3] Present address: Oxford NanoImaging, Oxford, United Kingdom

Content:

Supplementary Figure 1: Single-molecule interactions of CbAgo:siDNA at different conditions

Supplementary Figure 2: Single-molecule interactions of CbAgo with guide 4, 5, 6 and tandem target (22 nt separation)

Supplementary Figure 3: Example of HMM software applied to data trace

Supplementary Figure 4: Triple target assay, Y-fork assay and Lin28 assay

Supplementary Figure 5: Interactions of CbAgo with the dsDNA block construct

Supplementary Figure 6: Example shuttling traces for 11 nt, 15 nt, 18 nt, 29 nt, 36 nt, 50 nt and 120 nt target separation

Supplementary Figure 7: Cartoon representation of target search mechanisms

Supplementary Figure 8: Cartoon representation of Ago search model

Supplementary Figure 9: Coulombic surface coloring of *Clostridium butyricum* Argonaute (CbAgo)

Supplementary Note 1: Binding times single-target including recapture events follow single-exponential distribution

Supplementary Note 2: Shuttling rate due to sliding alone

Supplementary Note 3: Shuttling rate triple-target construct

Supplementary Note 4: Error estimates using bootstrapping

Supplementary Table 1: Shuttling times of two target DNA constructs for different distances

Supplementary Table 2: Oligonucleotides used for this study

Supplementary Figure 1

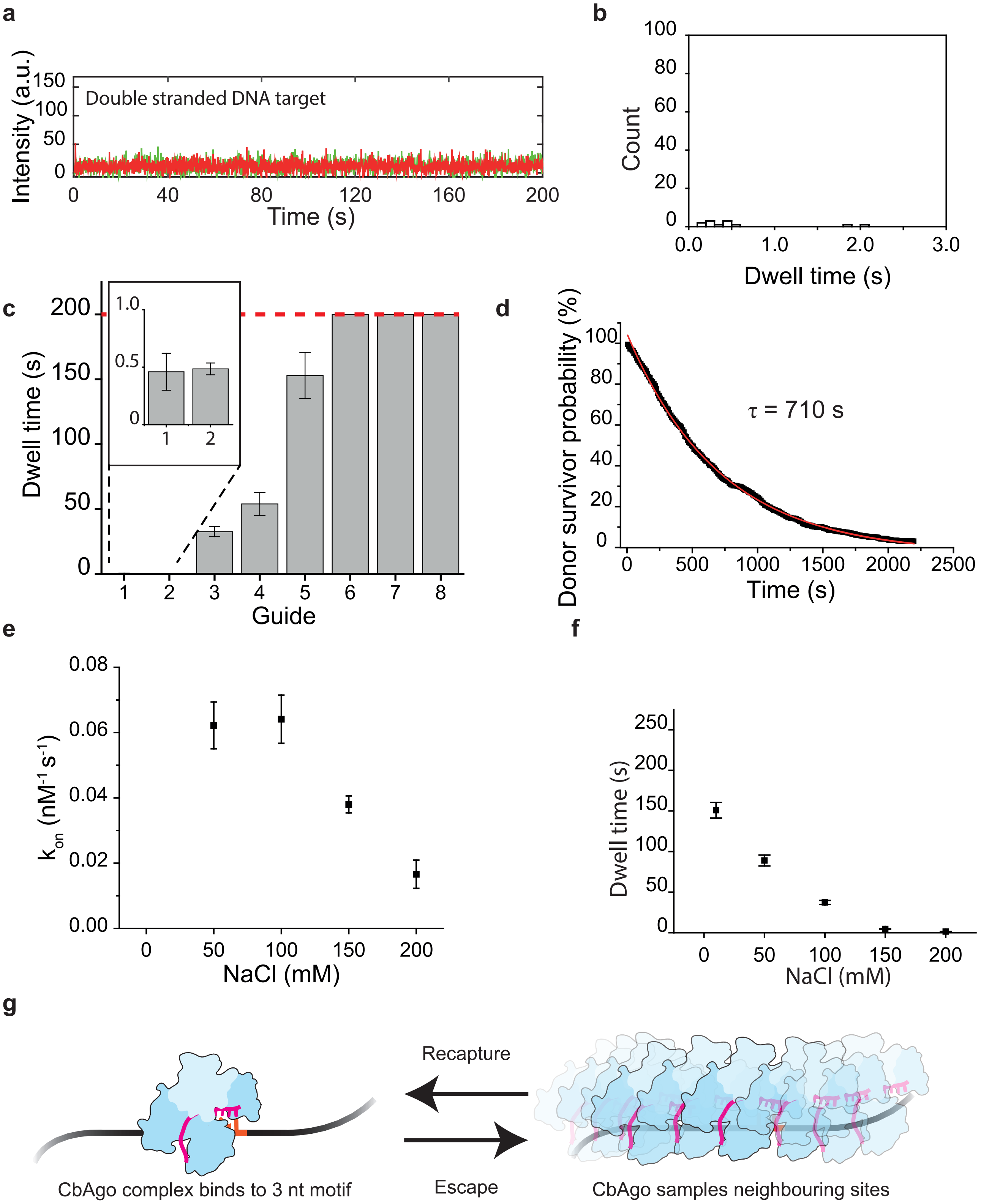

Supplementary Figure 1. Single molecule interactions of CbAgo:siDNA (2-4 nt) at different conditions.
(a) Representative trace single-molecule interaction of CbAgo-siDNA (let7) with full target dsDNA target immobilized on the surface (~300 per FoV). Exposure time is 100 ms.
(b) Dwelltime distribution of CbAgo-guide 3-dsDNA target interactions. Number of molecules recorded n = 540. Number of datapoints n = 12.
(c) Average dwell time of protein bound to target versus guide length for N=1 to N=8. The error bars are taken from the 95% confidence interval of bootstrapped dwelltimes (20,000 empirical bootstraps). The striped red line indicates the observation time, limited by photobleaching.
(d) Survival plot of donor only (Cy3) constructs in standard experimental conditions (100 mM NaCl, 50 mM Tris-HCl pH 8.0). Mean donor bleaching time was obtained by a single exponential fit to the survival probability plot.
(e) Binding rate for different salt concentrations for N=3 (nt 2-4) between guide and single target.
(f) Dwell time of CbAgo and a single-stranded single target DNA construct (N=3) at 10, 50, 100, 150 and 200 mM NaCl concentration. Total measurement time = 250 s. Error bars are indicating the 95% percentile of 20,000 empirical bootstraps of the mean dwell time.
(g) Schematic image indicating the dynamic escape and recapture events of CbAgo. Source data are provided as a source data file

Supplementary Figure 2

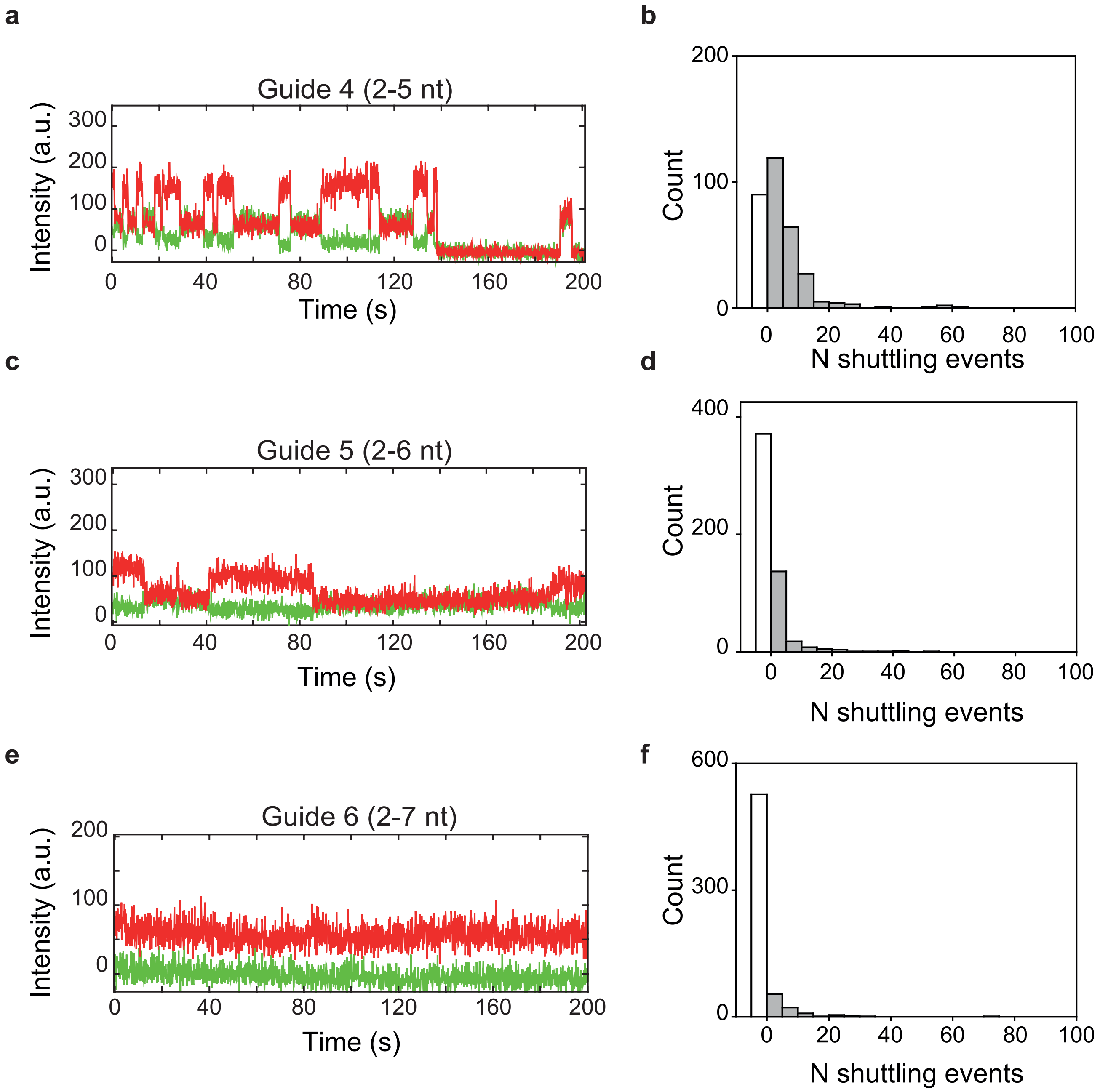

Supplementary Figure 2. Single-molecule interactions of CbAgo with guide 4, 5, 6 and tandem target (22 nt separation).
(a) Representative trace of binding events by CbAgo with guide 4 (nt 2-5). Duration of observation 200 s.
(b) Shuttling event distribution for guide 4 (nt 2-5). Bin size = 5. The white bar represents binding (no shuttling) events followed by dissociation. N = 317.
(c) Representative trace of binding events by CbAgo with guide 5 (2-6).
(d) Shuttling event distribution for guide 5 (2-6 nt). Bin size = 10. The white bar represents events that consists of single molecule binding followed by dissociation. n = 550.
(e) Representative trace of guide 6 (2-7 nt) interaction.(f) Shuttling event distribution for guide 6. The white bar represents events that consists of single molecule binding followed by dissociation. n = 621.
Source data are provided as a source data file

Supplementary Figure 3

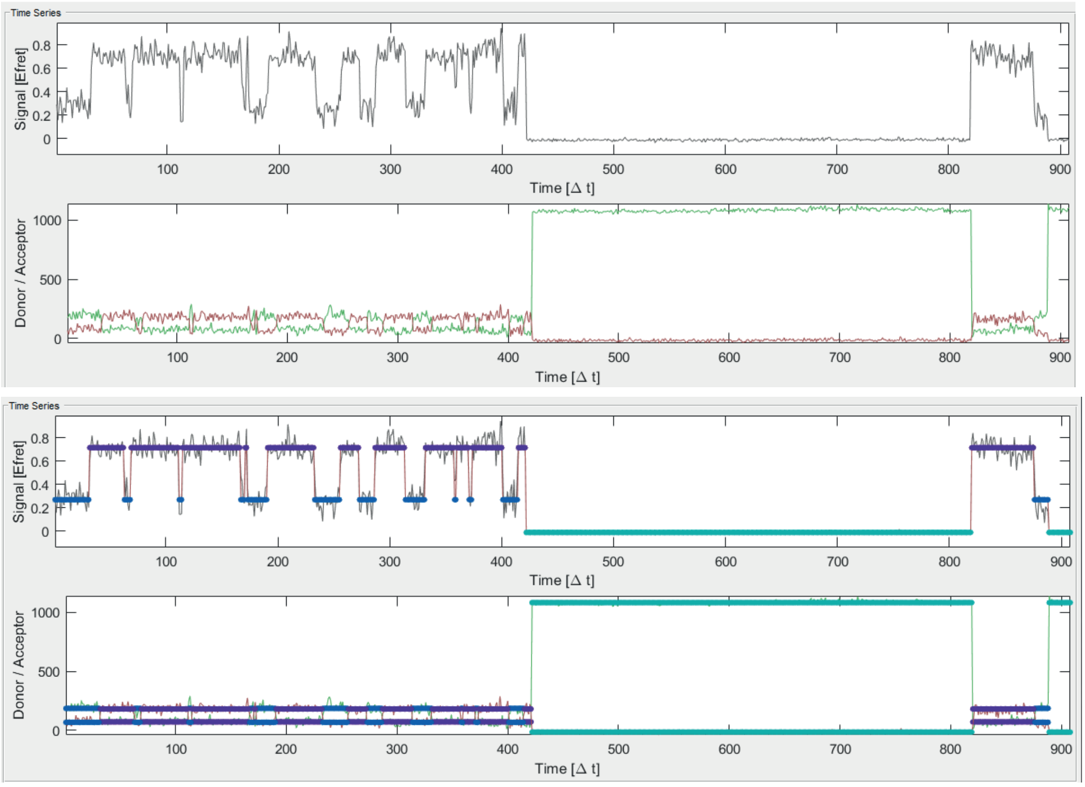

Supplementary Figure 3. Example of HMM software applied to data trace.
(Top) An example shuttling trace of CbAgo in the user interface of ebFRET. The donor and acceptor intensities plotted versus time. The donor intensity is enhanced artificially in absence of any signal, resulting in an extra zero FRET state (upper subfigure).
(Bottom) The donor, acceptor and FRET intensities overlaid with states resulting from the Hidden Markov Modeling. The HMM analysis program recognizes the unbound state as an extra state (light blue), while low FRET and high FRET are respectively assigned dark blue and purple.

Supplementary Figure 4

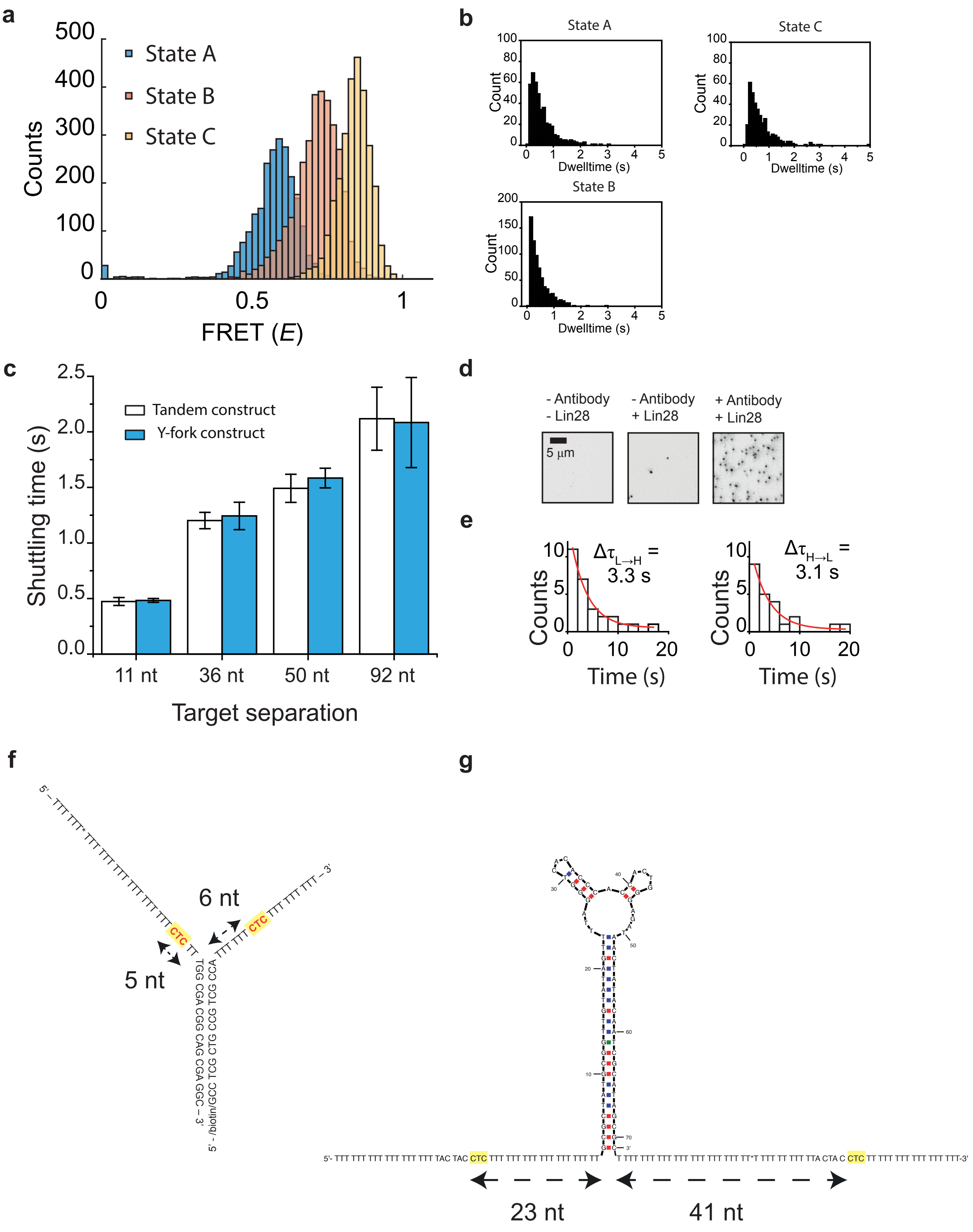

Supplementary Figure 4. Triple target assay, Y-fork assay and Lin28 assay

(a) FRET histogram of three-target assay. n = 168 molecules

(b) Dwell time histograms for respectively the low FRET, mid FRET and high FRET state of the three target assay.

(c) Shuttling rate of Y-fork constructs (blue) compared to tandem target assay (white) for 11 nt, 36 nt, 50 nt and 92 nt target separation. The error bars indicate the 95% percentile of 20,000 bootstrapped mean dwell times.

(d) An EMCCD image of the acceptor channel. (Left) In absence of Lin28 protein and antibody with Cy5 labeled DNA. (Middle) In absence of antibody, but in presence of Lin28 protein and Cy5 labeled DNA. (Right) In presence of antibody, Lin28 protein and Cy5 labeled DNA.

(e) Individual dwell times from low FRET state to high FRET state (left) and vice versa (right).

(f) Sequence schematic for the Y-fork 11 nt, indicating the target sites and their respective distances to the junction.

(g) Sequence schematic for the Lin28 blockade assay, indicating the target sites and their respective distances to the junction/protein. Source data are provided as a source data file

Supplementary Figure 5

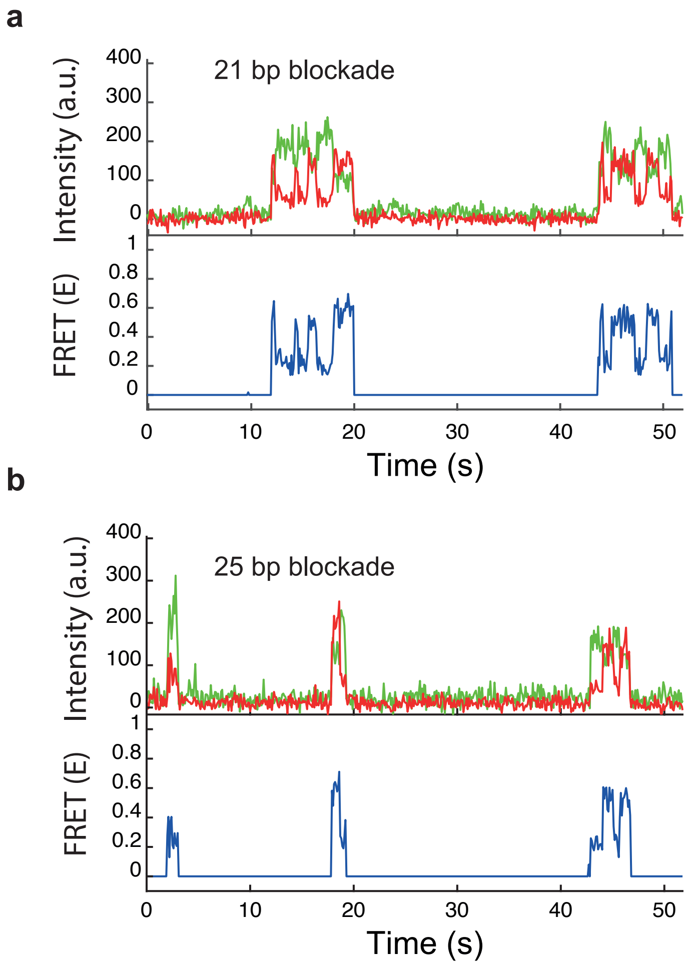

Supplementary Figure 5: Interactions of CbAgo with the dsDNA block construct.
(a) Representative trace of CbAgo interacting with a 21 bp DNA blockade construct.
(b) Representative trace of CbAgo interacting with a 25 bp DNA blockade construct.

Supplementary Figure 6

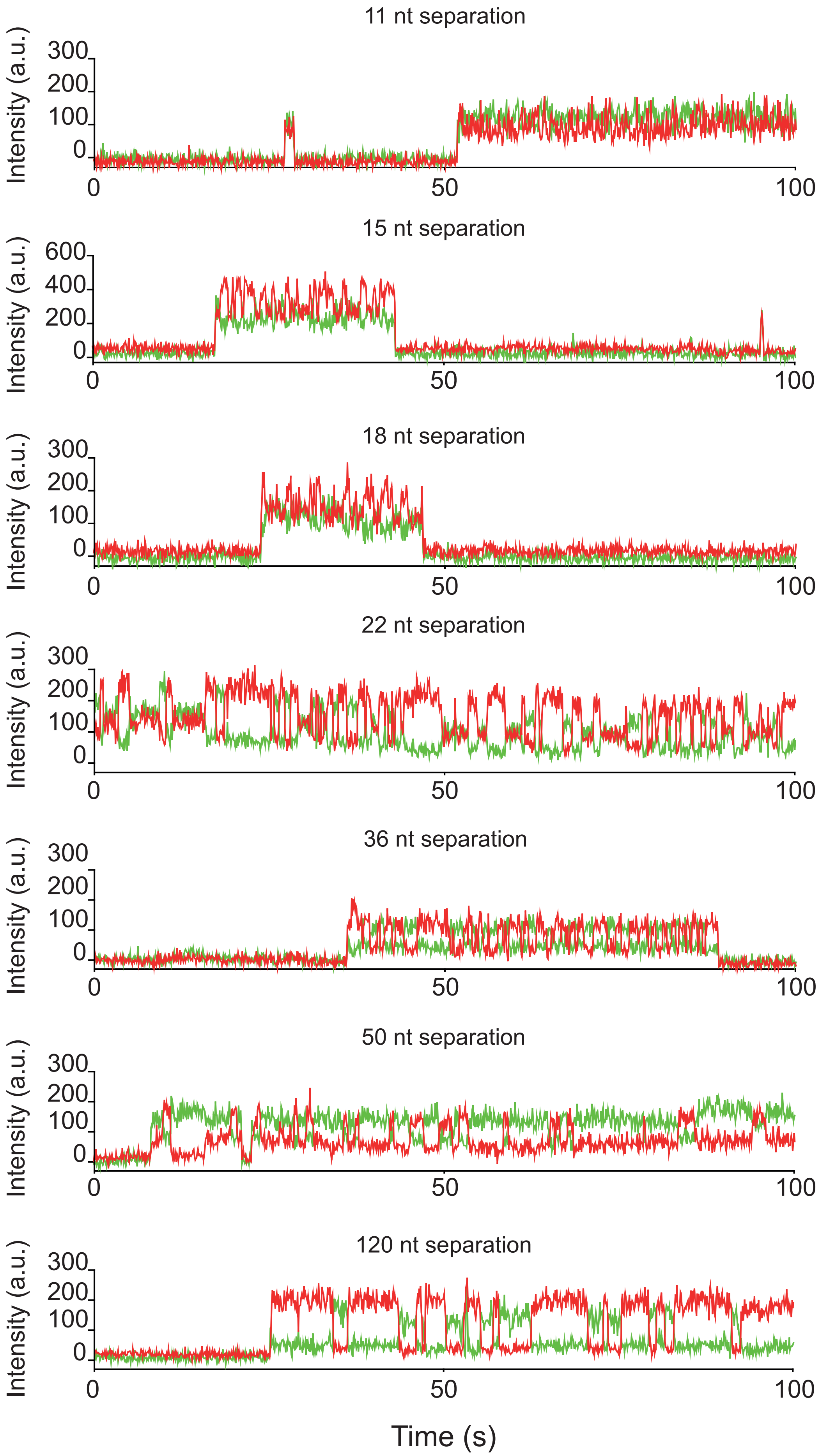

Supplementary Figure 6: Example shuttling traces for 11 nt, 15 nt, 18 nt, 22 nt, 29 nt, 36 nt, 50 nt and 120 nt target separation.

Supplementary Figure 7

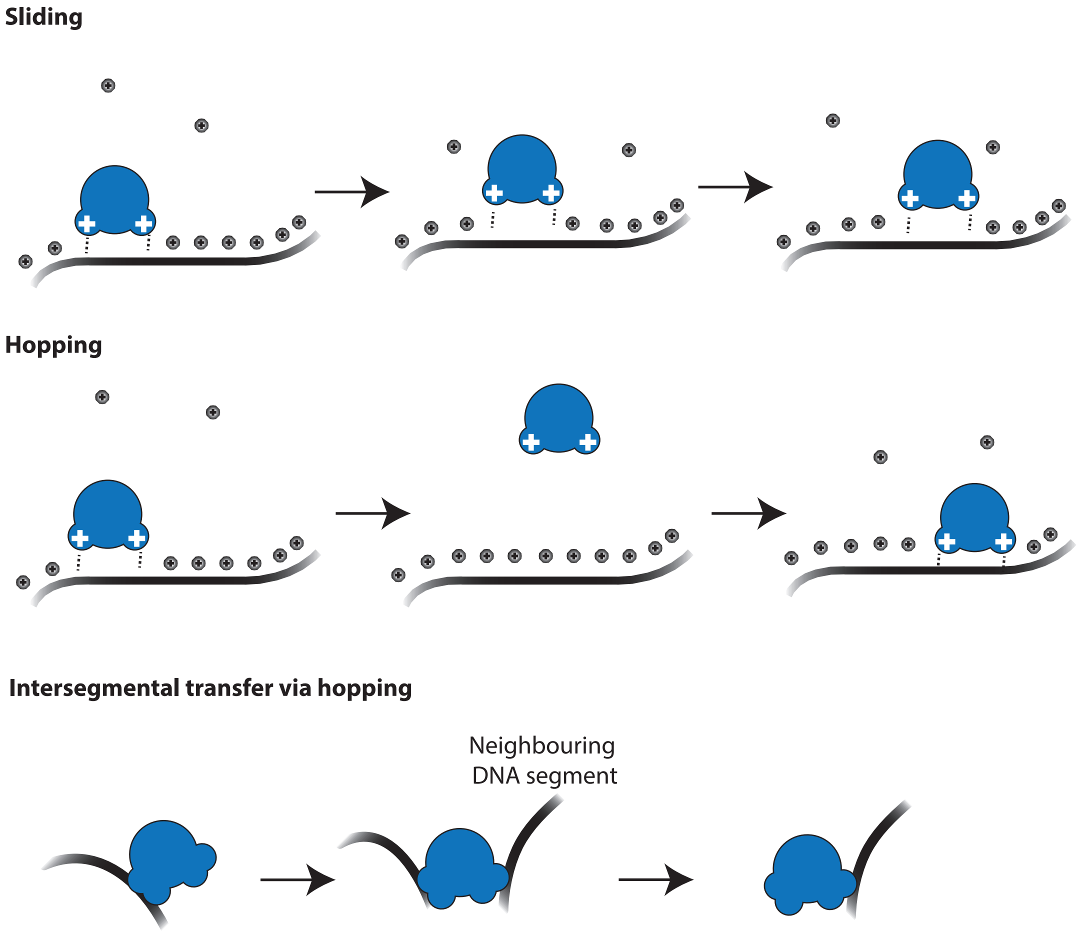

Supplementary Figure 7: Cartoon representation of target search mechanisms.
Sliding: Proteins that undergo sliding make a well-correlated movement along the contour of the nucleic acid substrate. There is no net displacement of counterions (grey circles).
Hopping: Proteins alternate quickly between a bound and unbound state with respect to DNA and there is counterion condensation upon dissociation of the protein. The method of diffusion is similar to 3D search, but its movements are correlated along the contour of the strand.
Intersegmental transfer: This mechanism is a specialized form of hopping where segments appear transiently close by allow the protein to transfer to this new segment.

Supplementary Figure 8

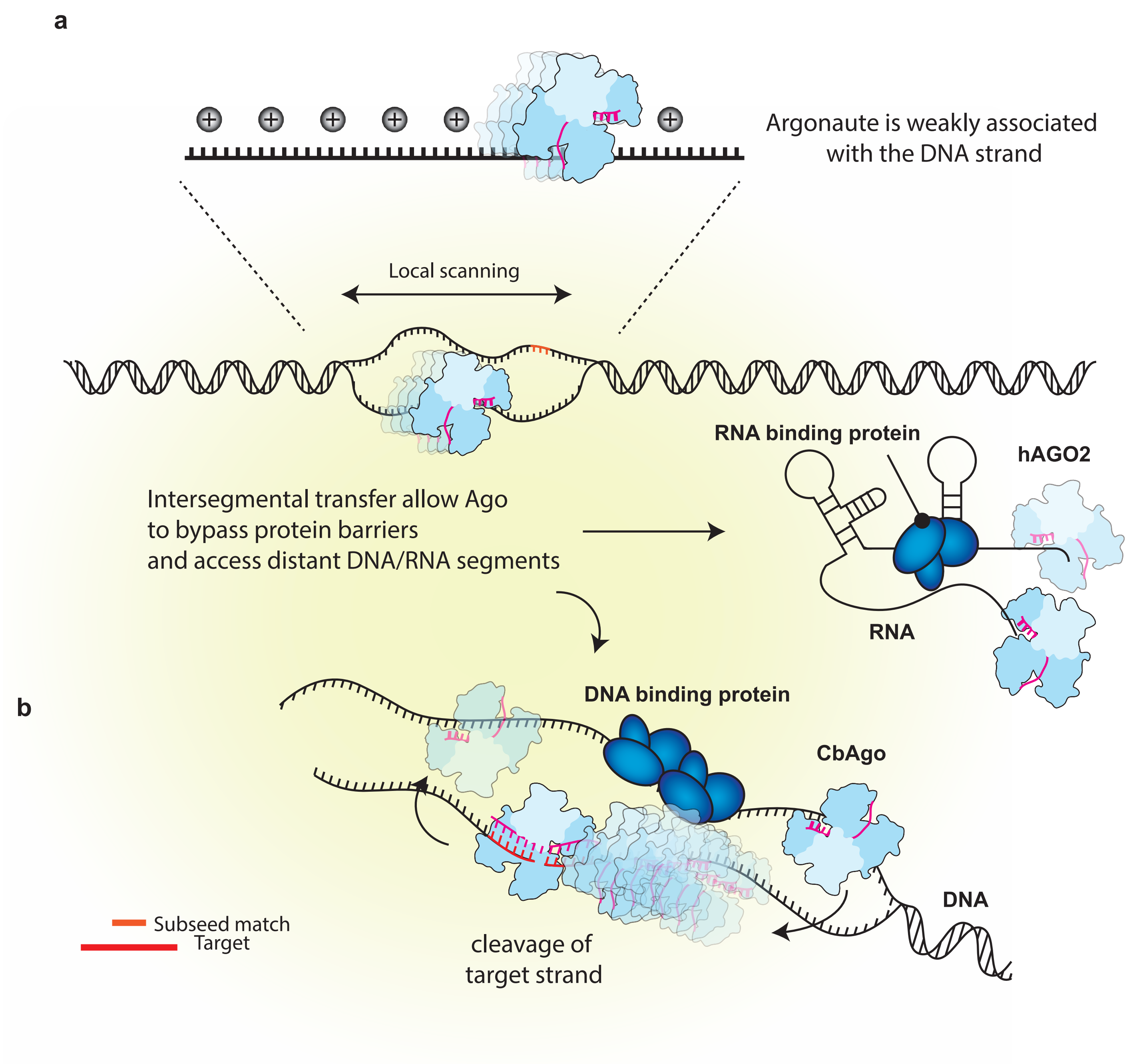

Supplementary Figure 8: Cartoon representation of Ago search model.
The Ago complex utilizes short transient interactions with nucleic acid strands to rapidly sample the adjacent (tens of nucleotides away) sites for possible targets. Loose interaction with the nucleic acid strand persists. Obstacles can be overcome through intersegmental transfer.

Supplementary Figure 9

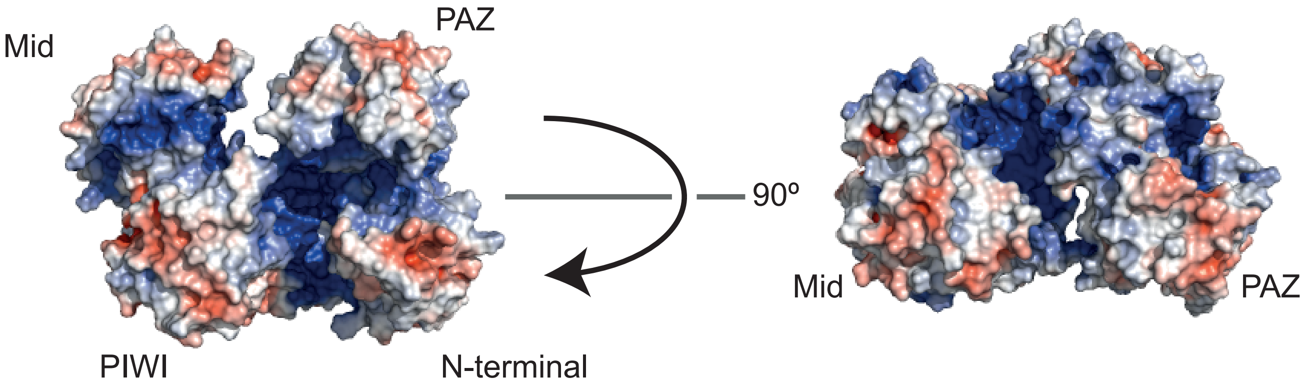

Supplementary Figure 9: Coulombic surface coloring of Clostridium butyricum Argonaute (CbAgo). The crystal structure of CbAgo (PDB 6qzk) (3.58 Å resolution) reveals the charge distribution. The cleft that contains the guide DNA and the target DNA is highly positively charged (blue).

Supplementary Note 1: Binding times single-target including recapture events follow single-exponential distribution

We here build a kinetic model for the lateral diffusion by CbAgo. Since Argonaute can in principle bind to any sequence along the DNA, we imagine the binding sites to be located a nucleotide apart. Further, we shall here only explicitly take sliding into account, which is represented as an unbiased random walk with unit step length. Assuming sliding should be a good approximation when considering only short distances traveled. If the protein is bound at the designed 3-nt sub-seed 'target' it can move to either of its neighbors at a rate of k_{esc} or unbind from the ssDNA at a rate of k_{ub}. When bound elsewhere movement and dissociation are assumed to happen instantaneously. To establish the manner in which these undetectable movements contribute to the observed dwell time distribution ($p_{\text{bound}}(\Delta t)$) we count all possible paths that the protein can take to dissociate following initial association to the sub-seed. In Laplace space the unbinding-time distribution, $P_{\text{ub}}(s) = \mathcal{L}\left\{p_{\text{bound}}(\Delta t)\right\}$, can be calculated as a product of the distributions of individual transitions (rather than their convolutions), summed over the possible paths towards unbinding. With an exponential distribution of stepping/escape times from the sub-seed trap,

$$p_{\text{esc}}(s) = \frac{2k_{\text{esc}}}{s + 2k_{\text{esc}} + k_{\text{ub}}} \qquad \text{(Supplementary Equation 1)}$$

, an unbinding time distribution from the trap

$$p_{\text{ub}}(s) = \frac{k_{\text{ub}}}{s + 2k_{\text{esc}} + k_{\text{ub}}} \qquad \text{(Supplementary Equation 2)}$$

and a probability to return, get recaptured at the trap, from either flank without unbinding P_{retrap} we can write

$$\begin{aligned} P_{\text{ub}}(s) &= \sum_{m=0}^{\infty} \left(p_{\text{esc}}(s) P_{\text{retrap}}\right)^m \left[p_{\text{ub}}(s) + p_{\text{esc}}(s)(1 - P_{\text{retrap}})\right] \\ &= \frac{k_{\text{ub}} + 2k_{\text{esc}}(1 - P_{\text{retrap}})}{s + k_{\text{ub}} + 2k_{\text{esc}}(1 - P_{\text{retrap}})} \end{aligned} \qquad \text{(Supplementary Equation 3)}$$

The sum on the left hand side of **Supplementary Equation 3** therefore accounts for the protein escaping from, and getting recaptured at the target an arbitrary amount of times (see **additional Supplementary Figure 1** below). The two terms outside the sum represent the probability distributions to unbind from either the target directly or after having escaped one final time respectively (**additional Supplementary Figure 1** below). Taking the inverse Laplace transform, we derive the observed dwell time distribution.

$$\begin{aligned} p_{\text{bound}}(\Delta t) &= \mathcal{L}^{-1}\left\{\frac{k_{\text{ub}} + 2k_{\text{esc}}(1 - P_{\text{retrap}})}{s + k_{\text{ub}} + 2k_{\text{esc}}(1 - P_{\text{retrap}})}\right\} \\ &= (k_{\text{ub}} + 2k_{\text{esc}}(1 - P_{\text{retrap}}))e^{-(k_{\text{ub}} + 2k_{\text{esc}}(1 - P_{\text{retrap}}))\Delta t} \end{aligned} \qquad \text{(Supplementary Equation 4)}$$

Hence, despite the multitude of possible bound states along the DNA the protein can reside in, the observed distribution remains single-exponential. The apparent dissociation rate follows

$$k_{\text{ub}}^{\text{observed}} = k_{\text{ub}} + 2k_{\text{esc}}(1 - P_{\text{retrap}}) \qquad \text{(Supplementary Equation 5)}$$

Given the assay selects for events that get (re-)captured, the observed rate is greater than its intrinsic value.

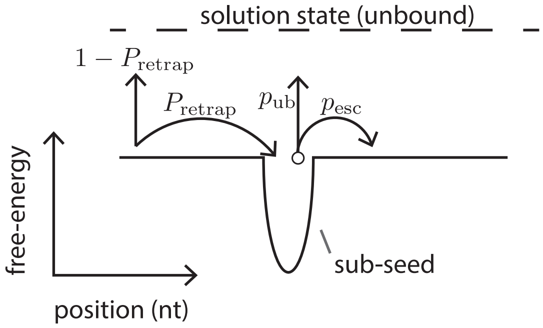

additional Supplementary Figure 1: *This figure illustrates how to construct* ***Supplementary Equation 3****. Starting from the sub-seed, Ago can either unbind directly (probability p_{ub}) or slide onto the non-specific binding sites flanking the trap (probability p_{esc}). When non-specifically bound, Ago can either laterally diffuse back into the sub-seed (probability P_{retrap}), or unbind (probability $1 - P_{retrap}$).*

Supplementary Note 2: Shuttling rate due to sliding alone

We seek to explain to what extend sliding contributes to the observed shuttling rate from the tandem-target assay. Given under the current experimental conditions about 13 shuttle events occur prior to unbinding, we shall ignore unbinding in the following analysis ($k_{\text{ub}} \ll k_{\text{esc}}$). To get the distribution of shuttle times ($p(\Delta t_{\text{shuttle}})$) we count all possible paths that lead the protein from one sub-seed to the other. If the two 3-nt nucleotide long sub-seeds are separated by $x_{\text{poly}-\text{T}}$ thymine nucleotides, the shuttle times are distributed as (setting $x_{\text{target}} = x_{\text{poly}-\text{T}} + 3 \geq 3$) (see **additional Supplementary Figure 2** below).)

$$P_{\text{shuttle}}(s, x_{\text{target}}) = \sum_{m=0}^{\infty} \left(p_{\text{esc}}(s) \left(\frac{1}{2} \times 1 + \frac{1}{2} \times P_{\text{R}}(x_{\text{target}}) \right) \right)^m p_{\text{esc}}(s) P_{\text{S}}(x_{\text{target}}) = \frac{k_{\text{esc}} P_{\text{S}}(x_{\text{target}})}{s + k_{\text{esc}} P_{\text{S}}(x_{\text{target}})}$$

(Supplementary Equation 6)

The two terms within the sum shown above represent recapture events at the initial trap via either the the flanking sequence (from which it always returns) or the poly-T stretch in between the traps (from which it returns with a probability $P_{\text{R}}(x_{\text{target}})$ without shuttling) (**additional Supplementary Figure 2** shown below). Finally, the term outside the sum accounts for successful shuttling events (which occurs with probability $P_{\text{S}}(x_{\text{target}}) = 1 - P_{\text{R}}(x_{\text{target}})$). Once the protein has left the initial trap $P_{\text{R}}(x)$ and $P_{\text{S}}(x)$ denote the distributions for either returning back to the initial trap or shuttling/making it across to the other, if the two traps are x nucleotides apart (see **additional Supplementary Figure 3** below)). Inverting the Laplace transformation of **Supplementary Equation 6** we obtain

$$\begin{aligned} p(\Delta t_{\text{shuttle}}) &= \mathcal{L}^{-1} \left\{ \frac{k_{\text{esc}} P_{\text{S}}(x_{\text{target}})}{s + k_{\text{esc}} P_{\text{S}}(x_{\text{target}})} \right\} \\ &= k_{\text{esc}} P_{\text{S}}(x_{\text{target}}) e^{-(k_{\text{esc}} P_{\text{S}}(x_{\text{target}}) \Delta t_{\text{shuttle}})} \end{aligned}$$

(Supplementary Equation 7)

Hence, the observed dwell time distributions are indeed single exponential. In terms of the microscopic model the average time is set by the escape rate from the trap modified by the probability to make it across once outside of it ($P_{\text{S}}(x_{\text{target}})$).
The probabilities P_{R} and P_{S}, for a given inter-trap distance x_{target} follow (see **additional Supplementary Figure 3** below)

$$P_{\text{R}}(x_{\text{target}}) = \sum_{m=0}^{\infty} \left(\frac{1}{2} P_{\text{R}}(x_{\text{target}} - 1) \right)^m \frac{1}{2}$$

(Supplementary Equation 8)

$$P_{\text{S}}(x_{\text{target}}) = \sum_{m=0}^{\infty} \left(\frac{1}{2} P_{\text{R}}(x_{\text{target}} - 1) \right)^m \frac{1}{2} P_{\text{S}}(x_{\text{target}} - 1)$$

(Supplementary Equation 9)

- from which we can write the recurrence relation

$$P_{\mathrm{S}}(x_{\mathrm{target}}) = P_{\mathrm{R}}(x_{\mathrm{target}})P_{\mathrm{S}}(x_{\mathrm{target}} - 1) \qquad \text{(Supplementary Equation 10)}$$

Using $(P_{\mathrm{S}}(x_{\mathrm{target}}) = 1 - P_{\mathrm{R}}(x_{\mathrm{target}}))$ the above can be re-written as

$$P_{\mathrm{S}}(x_{\mathrm{target}}) = \frac{P_{\mathrm{S}}(x_{\mathrm{target}} - 1)}{P_{\mathrm{S}}(x_{\mathrm{target}} - 1) + 1} \qquad \text{(Supplementary Equation 11)}$$

which subjected to the boundary condition $P_{\mathrm{S}}(1) = 1$ - signifying that if the traps are placed adjacent to each other, the shuttle is complete once the protein escaped the initial trap - has the simple solution

$$P_{\mathrm{S}}(x_{\mathrm{target}}) = \frac{1}{x_{\mathrm{target}}} \qquad \text{(Supplementary Equation 12)}$$

Taken together, the observed shuttling time equals

$$\Delta\tau_{\mathrm{shuttle}} = \frac{1}{k_{\mathrm{esc}}P_{\mathrm{S}}(x_{\mathrm{target}})} = \frac{x_{\mathrm{target}}}{k_{\mathrm{esc}}} \qquad \text{(Supplementary Equation 13)}$$

Note that $x_{\mathrm{target}} \geq 3$, as the two sub-seeds cannot overlap. A fit of **Supplementary Equation 13** to the experimental data for x_{target} of 11nt, 15nt, 18nt and 22nt in Figure 3 of the main manuscript were used to estimate the value of k_{esc} for CbAgo.

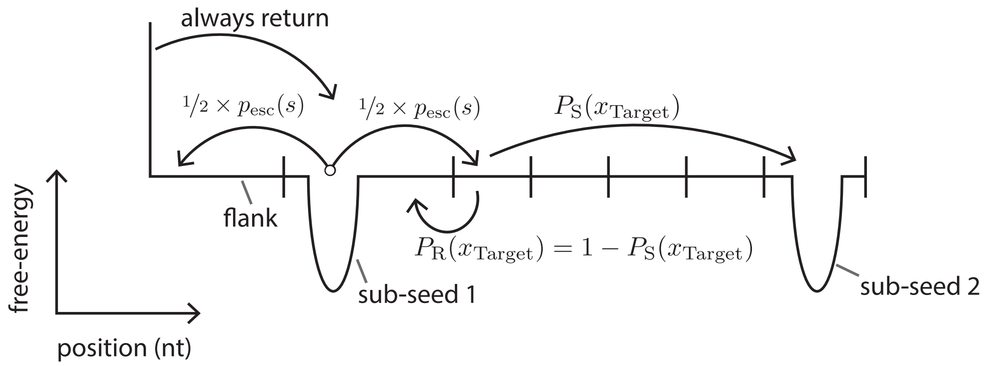

additional Supplementary Figure 2: *This figure illustrates how to construct* ***Supplementary Equation 6****. Ago slides to either of its neighboring sites with equal probability. Every shuttle event starts with Ago bound to one of the sub-seed sequences. After residing there for a time distributed as $p_{esc}(s)$, half of the times Ago moves onto the flank (from which it always returns by assumption), while the other half of the times the protein slid onto the poly-T sequence in between the two sub-seeds. All movements along these intermediate sites occur too fast to observe, which is why we only take into account to probability $P_S(x_{\mathrm{target}})$ of completing the shuttle event when x_{target} sites separate Ago from the second sub-seed.*

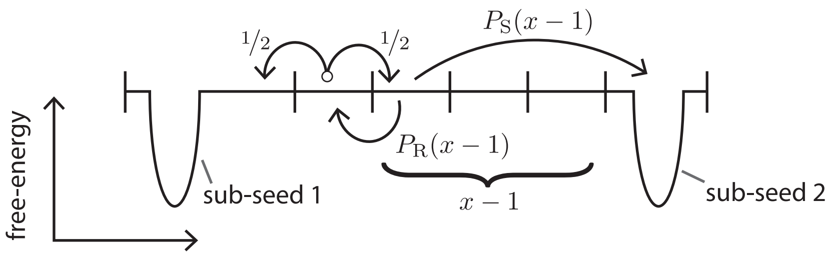

additional Supplementary Figure 3: *This figure illustrates how to construct* ***Supplementary Equation 8*** *and* ***Supplementary Equation 9****. Let $P_S(x)$ denote the probability to complete the shuttle when x sites separate Ago from the second sub-seed. Ago walks to either of its neighboring sites with equal probability. Therefore, when situated next to the first sub-seed, Ago gets recaptured half of the times it makes a move, while the other half has a probability of $P_S(x-1)$ to result in a completed shuttle event.*

Supplementary Note 3: Shuttling rate triple-target construct

For the assay using three sub-seed targets, we can now predict both the time needed to slide from any of the outer ones to the inner ($C \rightarrow B$) and the average time needed to slide along the opposite path ($B \rightarrow C$). The former is equal to the time measured on the tandem target construct, denoted above as $\Delta t_{\mathrm{shuttle}}$ (**Supplementary Equation 13**, $\Delta\tau_{\mathrm{CB}} = \Delta t_{\mathrm{shuttle}}$). We obtain $\Delta\tau_{\mathrm{BC}}$, via the distribution of lifetimes in the middle trap

$$P(\text{leave } B|\text{arrive at } C)(t) = \frac{P(\text{leave } B)(t)}{P(\text{arrive at } C(\text{and not } A))} \quad \text{(Supplementary Equation 14)}$$

Using that the distance between A and B is equal to that in between B and C, in Laplace space, the time spent at target B is distributed as ($P_{\mathrm{B}}(t) \equiv P(\text{leave } B)(t)$)

$$P_{\mathrm{B}}(s, x_{\mathrm{target}}, k_{\mathrm{esc}}) = \sum_{m=0}^{\infty}\left(\frac{1}{2}p_{\mathrm{esc}}(s) \times 2 \times P_{\mathrm{R}}(x_{\mathrm{target}})\right)^m \frac{1}{2}p_{\mathrm{esc}}(s)P_{\mathrm{S}}(x_{\mathrm{target}})$$

(Supplementary Equation 15)

The sum accounts for all paths that return to target B. Given the equal distances between all targets on the construct the probability to not make it across to either A or C are equal, which gives rise to the factor of two. The factor outside the sum accounts for the fact that the protein must eventually leave B and make it across to either A or C. Using the same technique as shown above, the average time spent in B equals

$$\tau_{\mathrm{B}}(x_{\mathrm{target}}) = \frac{x_{\mathrm{target}}}{4k_{\mathrm{esc}}} \quad \text{(Supplementary Equation 16)}$$

Using that half of the times the protein arrives at A, rather than C, results in the average dwell-time/shuttling time conditioned on moving from B to C (using eq. **Supplementary Equation 14**):

$$\Delta\tau_{\mathrm{BC}}(x_{\mathrm{target}}) = 2\tau_{\mathrm{B}}(x_{\mathrm{target}}) = \frac{x_{\mathrm{target}}}{2k_{\mathrm{esc}}} \quad \text{(Supplementary Equation 17)}$$

Supplementary Note 4: error estimates using bootstrapping

Fitting the data from the tandem target assay to **Supplementary Equation 13** provides the estimate of k_{esc}. We bootstrapped the dwell time distributions acquired using the original tandem target assay (distances of 11nt, 15nt, 18nt and 22nt). For each of the 10^5 bootstrap samples we calculated new values for the associated $\Delta t_{\mathrm{shuttle}}$'s and repeated the fit to **Supplementary Equation 13** to obtain an error estimate in the fitted value of the escape rate.

After using the data from the tandem target assay to estimate k_{esc} there are no more free parameters remaining when predicting the data for the triple-target assay. Performing the bootstrap procedure for k_{esc}, and using **Supplementary Equation 13** and **Supplementary Equation 17** results in the 95% confidence intervals shown in figure 4D in the main manuscript.

An error estimate for the experimental values of $\Delta\tau_{\mathrm{BC}}$ and $\Delta\tau_{\mathrm{CB}}$ were obtained using 10^5 bootstrap samples of the dwell time distributions measured using the triple-target assay. All analysis was performed with a custom code written in Python.

Supplementary Table 1. Shuttling times of two target DNA constructs for different distances. The upper bound and lower bound are estimated through 20000 bootstraps of the acquired dwell times. Related to Figure 3.

Target distance (nt)	Lifetime (sec)	Lower bound lifetime (sec)	Upper bound lifetime (sec)	Shuttling rate (sec^{-1})	Lower bound shuttling rate (sec^{-1})	Upper bound shuttling rate (sec^{-1})
11	0.47	0.46	0.49	2.11	2.04	2.19
15	0.83	0.81	0.87	1.19	1.15	1.24
18	1.17	1.11	1.24	0.85	0.81	0.90
22	1.79	1.74	1.86	0.56	0.54	0.57
29	1.36	1.30	1.42	0.73	0.70	0.77
36	1.19	1.16	1.23	0.84	0.81	0.86
50	1.52	1.46	1.57	0.66	0.64	0.68
64	1.65	1.59	1.71	0.61	0.59	0.63
92	1.94	1.85	2.02	0.52	0.49	0.54
120	2.11	2.03	2.19	0.47	0.46	0.49

Supplementary Table 2. Oligonucleotides used for this study. /5Phos/ indicates the phosphorylated 5’ end. /iAmMC6T/ indicates a thymine with an amine group attached through a carbon 6 linker. /Biotin dT/ indicates the biotin placed on the back of thymine.

**Name Oligo**	**Sequence 5’-> 3’**	**Length (nt)**
**GUIDE**		
Guide 3nt (2-4)	5- /5Phos/CGA GTA TT/iAmMC6T/ TTT TTT TTT TTT T – 3’	22
Guide 4nt (2-5)	5'-/5Phos/CGA GGA TT/iAmMC6T/ TTT TTT TTT TTT T - 3'	22
Guide 5nt (2-6)	5'- /5Phos/CGA GGT TT/iAmMC6T/ TTT TTT TTT TTT T - 3'	22
Guide 6nt (2-7)	5'- /5Phos/CGA GGT AT/iAmMC6T/ TTT TTT TTT TTT T - 3 '	22
Guide 7nt (2-8)	5'- /5Phos/CGA GGT AGA /iAmMC6T/TT TTT TTT TTT T -3'	22
Guide 8nt (2-9)	5'- /5Phos/ CGA GGT AG/iAmMC6T/ TTT TTT TTT TTT T - 3 '	22
**TARGET**		
8nt tandem target 7nt separation	5' - TTT TTT TTT TTT TTT TTT CTC TTT TCT CT/iAmMC6T/ TTT TTT TTT TTT TTT TTT TTT TTT TTT T/biotin/ -3'	58
8nt tandem target 11nt separation	5' - TTT TTT TTT TTT TTT TTT CTC TTT TTT TT CT CT/iAmMC6T/ TTT TTT TTT TTT TTT TTT TTT TTT TTT T/biotin/ -3'	62
8nt tandem target 15nt separation	5' - TTT TTT TTT TTT TAC TAC CTC TTT TTT TA CTA CCT CT/iAmMC6T/ TTT TTT TTT TTT TTT TTT TTT TTT TTT T/biotin/ -3'	66
8nt tandem target 18nt separation	5' - TTT TTT TTT TTT TAC TAC CTC TTT TTT TTT TA CTA CCT CT/iAmMC6T/ TTT TTT TTT TTT TTT TTT TTT TTT TTT T/biotin/ -3'	69
8nt tandem target 22nt separation	5' - TTT TTT TTT TTT TAC TAC CTC TTT TTT /iAmMC6T/TT TTT TTA CTA CCT CTT TTT TTT TTT TTT TTT TTT TTT TTT TTT T/biotin/ -3'	73
8nt tandem target 29nt separation	5' –TTT TTT TTT TTT TA CTA CCT CTT TT TTT TT/iAmMC6T/ TTT TTT TTT TTA CTA CCT CTT TTT TTT TTT TTT TTT TTT TTT TTT TTT TT/biotin/-3'	81
8nt double target 36nt separation	5' –TTT TTT TTT TTT TTA CTA CCT CTT TTT TTT TTT TTT TT/iAmMC6T/ TTT TTT TTT TTA CTA CCT CTT TTT TTT TTT TTT TTT TTT TTT TTT TTT TT/biotin dT//Phos/-3'	89
8 nt tandem target 50nt separation	5' –TTT TTT TTT TTT TTA CTA CCT CTT TTT TTT TTT TTT TTT TTT TT TTT TTT TT/iAmMC6T/ TTT TTT TTT TTA CTA CCT CTT TTT TTT TTT TTT TTT TTT TTT TTT TTT TT/biotin dT//Phos/-3'	104
8 nt tandem target 64 nt separation	5' –TTT TTT TTT TTT TTA CTA CCT CTT TTT TTT TTT TTT TTT TTT TTT TTT TTT TTT TTT TTT TTT T/iAmMC6T/T TTT TTT TTT TTT ACT ACC TCT TTT TTT TTT TTT TTT TTT TTT TTT TTT TT/biotin-dT/ /Phos/-3'	117
8 nt tandem target 92 nt separation	5' –TTT TTT TTT TTT TTA CTA CCT CTT TTT TTT TTT TTT TTT TTT TTT TTT TTT TTT T TTT TTT TTT TTT TTT TT TTT TTT TTT TTT TTT TTT TTT T/iAmMC6T/T TTT TTT TTT T ACT ACC TCT TTT TTT TTT TTT TTT TTT TTT TTT TTT TT/biotin-dT/ /Phos/-3'	145
8nt double target 120nt separation	5' –TTT TTT TTT TTT TTA CTA CCT CTT TTT TTT TTT TTT TTT TTT TTT TTT TTT TTT TTT TTT TTT TTT TTT TTT TTT TTT TTT TTT TTT TTT TTT TTT TTT TTT TTT TTT TTT TTT TT/iAmMC6T/ TTT TTT TTT TTA CTA CCT CTT TTT TTT TTT TTT TTT TTT TTT TTT TTT TT/biotin dT//Phos/-3'	171
11nt Y-fork	5’ – TTT TTT* TTT TTT TTT TTT TTT TTT CTC TT TGG CGA CGG CAG CGA GGC – 3’	47
11nt Y-fork biotin	5’ - /biotin/GCC TCG CTG CCG TCG CCA TTT TTT CTC TTT TTT TTT – 3’	36
50nt Y-fork	5‘- TTT TTT TTT TTT* TTT TTT TAC TAC CTC TTT TTT TTT TTT TTT TT TTT TGG CGA CGG CAG CGA GGC – 3‘	65
Y-fork stem (not for Y11)	5’ – /biotin/GCC TCG CTG CCG TCG CCA TTT TTT TTT TTT TTT TTT TTT TAC TAC CTC TTT TTT TTT – 3’	57

36nt dsDNA target	5' – TTT TTT TTT TTT T TA CTA C CTC T CGG ACC AAC AGC GGG /T-biotin/AC GGC TGT GC TA CTA CCT CTT TTT TTT TTT TTT TTT TTT - 3'	78
36nt dsDNA block v2 3' biotin	5' –TTT TTT TTT TTT T TA CTA C CTC T CGG ACC AAC AGC GGG TAC GGC TGT GC TA CTA CCT CTT TTT TTT TTT TTT TTT TTT TTT TTT TTT TT/biotin dT/- 3'	91
36nt dsDNA block 5'end truncated	5' –CTA C CTC T CGG ACC AAC AGC GGG TAC GGC TGT GC TA CTA CCT CTT TTT TTT TTT TTT TTT TTT TTT TTT TTT TT/biotin dT/- 3'	75
36nt 25nt block	5' - TA GC ACA GCC GT* A CCC GCT GTT GGT- 3'	25
36nt 21nt block	5'- GC ACA GCC GT* A CCC GCT GTT G- 3'	21
36nt 17nt block	5' -ACA GCC GT* A CCC GCT GT- 3'	17
Triple target	5' – T/iAmMC6T/ TTT TTT TTT TAC CTC TTT TTT ACC TCT TTT TTA CCT C TTT TTT TTT TTT TTT TTT TTT TTT TTT TTT/biotin/ -3'	69
No target DNA	5'- TTT TTT TTT TTT TTT TTT TTT TTT TTT /iAmMC6T/TT TTT TTT TTT TTT TTT TTT TTT TTT TTT TTT TTT TTT TTT TTT TGG CGA CGG CAG CGA GGC -3'	90
8nt single target	5' - TTT TTT TTT TTT TTT TTT TTT TTT TTT /iAmMC6T/TT TTT TTA CTA CCT CTT TTT TTT TTT TTT TTT TTT TTT TTT TTT T/biotin/-3'	73
3' biotin stem	5' - GCC TCG CTG CCG TCG CCA biotin – 3'	18
Lin28 double target	5'- TTT TTT TTT TTT TTT TTT TAC TAC CTC TTT TTT TTT TTT TTT TTT TTG CGC TAT GCG GTT GTA TAG TTT TAG GGT CAC ACC CAC CAC TGG GAG ATA ACT ATA CAA TCG CAT AGC GCT TTT TTT TTT TTT TTT TTT TTT T/iAmMC6T/T TTT TTT TTA CTA CCT CTT TTT TTT TTT TTT TTT-3'	174
