# Supplementary material for: Argonaute bypasses cellular obstacles without hindrance during target search
Source: Nat Commun. 2019 Sep 26;10:4390. doi: 10.1038/s41467-019-12415-y (PMC6763497; doi:10.1038/s41467-019-12415-y)
Supplement: Supplementary file 2 — Peer Review File [file 41467_2019_12415_MOESM2_ESM.pdf]

Reviewers' comments:

Reviewer #1 (Remarks to the Author):

Cui et a.

Argonaute bypasses cellular obstacles without hindrance during target search

In this manuscript, the authors have used single molecule FRET analysis to determine how CbAgo finds potential target sites. A weak, 3-nt target site was immobilized and incubated with CbAgo loaded with a complementary guide RNA. Using two distinct target sites on one DNA molecule, their findings support a lateral diffusion model, in which Ago proteins diffuse along the template without full dissociation. The authors extended their studies and calculated lateral diffusion rates. When three target sites are present, CbAgo preferentially diffuses to the closest one but occasionally also occupies the distal site. They further included barriers (a DNA-DNA junction by hybridizing two DNA strands to each other or Lin28b binding to a hairpin) between two target sites and their findings suggest that CbAgo performs intersegmental jumps to bypass these obstacles. Ionic strength (salt concentration) affects these longer distance jumps but local scanning is independent of the salt concentration. Thus, the authors suggest a model, in which local scanning is performed by a gliding mechanism whereas longer distance are passed by a mechanism involving the ionic cloud provided by salt ions.

This is a solid and well-performed study with novel insights and concepts on Ago protein-substrate interactions. It is a continuation of previous work of the same authors that presents validation of existing data but also novel findings. However, there are a number of issues that need to be clarified.

1. One of the major issues with the study is that a very weak target site has been chosen. Three nt complementary would not be sufficient to bind Ago proteins to target sites. Such 'targets' might be very frequent in any genome and Ago proteins would dwell for a very short time. Although interesting insights into general kinetics on such sites are revealed, it remains unclear where this is similarly relevant for targets with higher degree of complementarity.

2. For the distal sites, one would assume that CbAgo would not move towards the target site but it would rather pass by due to DNA flexibility. One could probably test this more directly. The target sites should be close enough to Lin28 in order to prevent that the sites can reach each other. In this case, only one single FRET signal should be observe. This would further strengthen the model.

3. What is a '3 nucleotide (nt) subseed motif...'. It is clear what it is, but why such a cryptic explanation? The remaining substrate is poly(T). Is there any particular reason why Ts have been selected? Could this also affect the outcome of the measurements?

4. References to Figure 1 in the results section might be incorrect. Isn't Figure 1d (bottom of page 5) actually 1f? 1e and 1h are not mentioned at all.

5. On page 6, the authors state that the two target sites are separated by 22 nt. Figure 2A shows a linker of 19 nts. Along the same lines, on page 8 they state that they chose neighboring targets similar to the target separation in the previous assays (11nt). Where do the 11 nt come from?

6. Reference to Hegge, Swarts et al. 2019 is incomplete.

Reviewer #2 (Remarks to the Author):

In this paper, Cui et al. use a combination of single-molecule FRET experiments and theoretical

modeling to interrogate the mechanism by which Argonaute proteins search for targets. They found that the Argonaute from C. butyricum (CbAgo) uses a new mode of lateral diffusion, which they termed "gliding", for short-range target searching. For long-range search, CbAgo employs intersegmental jumps to reach distant sites. This combination of target-scanning modes allows Ago to bypass obstacles without hindrance. In general, the experiments are cleverly designed, especially the usage of multiple adjacent target sites to derive the escape rate. The data and analyses are both of high quality. The novel mechanism discovered here may be broadly applicable to other nucleic acid-guided proteins.

However, the manuscript is somewhat sloppily written. The authors should take more care with the text, figures, and references in terms of accuracy and consistency. At times key data are missing without explanation. The following issues need to be addressed before the paper can be accepted for publication at Nature Communications.

Major points:
(1) The important distinction between "sliding" and "gliding" relies on the observation that CbAgo can bypass physical barriers. However, it is not entirely clear whether the Y-fork construct in Figure 5a really presents a barrier to CbAgo, since the ssDNA is essentially contiguous in this construct. The protein roadblock in Figure 5c more likely presents a barrier, but that also depends on how the two single strands come out of Lin28b. A much more effective construct is the one depicted in Supplementary Figure 5, which contains a stretch of dsDNA that disrupts the continuous ssDNA. The authors seem to have done this experiment in Supplementary Fig. 5d-h. But these data were never discussed. Was CbAgo still able to shuttle between the two sites with a dsDNA roadblock in between? In fact, Supplementary Figure 5 contains many results, but was only briefly mentioned once in the text (Line 203-205).
(2) The distinction between "gliding" and "hopping" relies on the "mild" versus "strong" dependence of the shuttling time on the ionic strength. What is the criterion used to define "mild" or "strong"? Is there a theoretical basis for this?
(3) The conclusion that CbAgo uses intersegmental jumps for target search across large distances mainly comes from the breakdown of the linear relationship between shuttling time and target separation at large distances shown in Figure 3. As the authors mentioned, such a mechanism relies on the ability of a distant segment of ssDNA to loop back into the proximity of CbAgo. It follows that the search efficiency should critically depend on the conformational entropy of the substrate, and should diminish on a force-stretched substrate (as demonstrated by Forget and Kowalczykowski, Nature 482:423-427 (2012)). Can the authors fix both ends of the ssDNA on surface and check whether CbAgo can still shuttle between two sites with a large separation? While such experiments are not required, the authors should at least discuss this possibility.
(4) The definition of "shuttling time" is confusing. I initially thought it represents the transition time between different FRET states. Then I realized that it actually represents the dwell time of each FRET state. Please specifically clarify this term or use another term.
(5) Instead of a supplementary table, a cartoon figure illustrating the different target search mechanisms (sliding, hopping, gliding, intersegmental jumping, etc.) and the combined searching mode of gliding and intersegmental jumping employed by CbAgo would be more informative. And I don't understand the difference between intersegmental jumping and intersegmental transfer.

Minor points:
(1) In Line 120-125, a single-exponential distribution is also consistent with stable binding to the target without any transient escape, whose lifetime would be governed by a single dissociation rate. How could the authors distinguish between the two scenarios? Is it because of the observed decrease in dwell time from 37 s to 1.7 s when changing from one target to two targets? Please comment.
(2) What are the dye positions for dual-target constructs with large distances (e.g., the 120-nt substrate in Fig. 3)? Can binding events to both sites still be detected and distinguished by FRET?
(3) In Figure 4d, the "shuttling times" for A-to-B and B-to-A events should be included.
(4) The shuttling time for 11-nt separation reported in Figure 3 and Figure 5b is not consistent.

(5) Instead of a categorically distinct substrate, the "no target" substrate in Figure 1c is actually a continuation of the series of substrates tested in Supplementary Figure 1c, with a guide length of N=1. The "T" in the poly(T) target sequence complements the "A" at nt #2 of the guide. The results for this substrate should be incorporate into Supplementary Fig. 1c for direct comparison. By the same token, a substrate with a guide length of N=2 would be nice to test too. Perhaps the authors already have the results?
(6) The figures are not arranged in the order in which they are referenced. In some cases the wrong figure is referred to. For example, in Line 187, "The exponential distribution of the dwell times (Supplementary Fig. 4c) suggests that…" should be Supplementary Fig. 4b. In Line 263, "…while our Y-fork experiments show that CbAgo need not be in constant physical contact with its substrate during this process (Figure 4b)." Does Fig. 4b tell us this?
(7) There are numerous typos throughout the manuscript (e.g., line 161, line 275, line 322).

Reviewer #3 (Remarks to the Author):

In "Argonaute bypasses cellular obstacles without hindrance during target search", Cui et al. present single molecule FRET (smFRET) experiments to elucidate the ssDNA target search mechanism of an Argonaute protein from the bacterium Clostridium butyricum (CbAgo). Through a series of single-molecule measurements on ssDNA with 1 to 3 target sequences, the authors determine that Ago identifies targets through facilitated diffusion, consisting of periods of lateral 1D diffusion over short distances with periods of intersegmental transfer, allowing Ago to bypass barriers such as DNA-bound proteins.

Overall this is a well-designed study on an important class of protein. The experiments are cleverly designed and the data convincing. However, there are important criticisms the authors need to address, as detailed below. The most important point is that the novelty of the results is unclear. Facilitated diffusion is a well-established mechanism of target search shown for a number of DNA binding proteins (transcription factors, restriction enzymes, etc.), and I am not convinced that the difference in dsDNA vs. ssDNA target search is that significant. I believe the authors overstate this point. While the quantitative model proposed is useful, the results themselves are unsurprising. Thus, it is unclear that the work rises to the level expected for publication in Nature Communications. The authors should address this point (and others enumerated below) before a recommendation on the manuscript can be made.

Specific points:
In their model, the shuttling time is proportional to the distance between target sites (Eq. (1)), which is corroborated by the data (Fig. 3). This seems counterintuitive, since a diffusive mechanism should lead to x^2 ~ t. Could the authors provide a simple explanation for this? Is this because the rate-limiting step is not the 1D diffusion itself but release of Ago from the binding site? This is not clearly explained in the text or SI.

As pointed out in the text (lines 227-228), for intersegmental transfer to occur, ssDNA must loop back into proximity of the bound Ago. The authors should be able to expand their model by quantitatively modeling the kinetics of intersegmental transfer. This can be done by accounting for the probability of looping, models for which can be found in the literature. Looping is highly dependent on DNA flexibility, and the authors could explicitly test their by introducing stiff dsDNA sections between target sites.

On a related point, the authors draw several conclusions from their measurements of search kinetics at different ionic strengths. However, DNA flexibility is also dependent on ionic strength. Is it possible that the changes in target search kinetics the authors observe upon increasing ionic strength are in fact due to changes in DNA flexibility, rather than changes in protein affinity to DNA (lines 243-249)? This alternative should be considered explicitly.

In several instances in the text, statements are made that are neither convincing nor supported by references. The authors need to address these:

Line 275-276: “CbAgo uses a target search mechanism distinct from other known DNA binding proteins.” As stated above in this review, facilitated diffusion has been exhibited in a host of other DNA binding proteins during target search. Even if one constrains oneself to ssDNA binding proteins, single-stranded DNA binding proteins (SSB) from E. coli have been shown to exhibit both 1D diffusion and intersegmental transfer. Therefore, this is not a new finding.

Line 276-277: “Since the CbAgo searches on ssDNA, not dsDNA, it cannot make use of the structural regularity of double stranded DNA.” This argument is made to highlight the difference between diffusion on ssDNA and dsDNA, but I do not find it compelling. It is unclear what evidence exists demonstrating that the structural regularity of dsDNA is essential for 1D diffusion. The authors need to motivate this statement better.

Line 327-328: “dsDNA is substantially more conducive for tight interactions than flexible single stranded nucleic acids”. I am not convinced this is true. Single-stranded DNA binding proteins (SSB) and recombinases RecA/Rad51 are examples of proteins that bind to ssDNA with high affinity. If anything, one could make the opposite argument, since the high flexibility of ssDNA would provide a low energy cost to deformation by protein binding, in contrast to dsDNA (e.g. exhibited by transcription factors, restriction enzymes).

In addition there are several typographical and grammatical errors in the text that need to be corrected:

Main text:
Line 155: “we kinetically model how the this time depends on…” should have one of the two articles, “the” or “this”, removed
Line 333: “orientate” should be “orient”

SI:
Section A, paragraph 1: “inital assocation” should be “initial association”
Section B, paragraph 2: “succesful” should be “successful”
Section B, paragraph 3: “accross” should be “across”
Section D, paragraph 2: “precedure” should be “procedure”

Reviewer #4 (Remarks to the Author):

This is a largely excellent manuscript that deals with an important subject -- namely, how -- in molecular detail -- do genome-regulatory proteins find their target sites on DNA? More specifically, and unlike the transcription factors that have largely been studied in this context in the past, the argonaute systems studied in this work operate by finding their targets on single-stranded (ss) DNA by use of a small nucleic acid guide component that is essential to final recognition of the target sequence. Thus these protein-nucleic acid complexes search the target by a multi-step process; first binding to the overall ssDNA target by non-sequence-specific electrostatic interactions with the protein component that permit various forms of 'lateral diffusion', and then recognizing the target by sequence complementarity with the nucleic acid guide component. The authors use a well-designed series of protein-nucleic acid labeled FRET constructs to study the rates and extents of translocation events in an attempt to define – at the molecular level -- how these argonaute complexes scan across and then recognize their ssDNA target sequences in the DNA genome.

The results obtained suggest that the overall search mechanism can be considered as the sum of two separate processes: (i) an electrostatically-stabilized general binding of the protein component of the argonaute complex to the overall ssDNA target on which it undergoes 'lateral diffusion' in the course of 'scanning' for the actual target site; and (ii) local recognition of the target sequence itself by complementary base-pairing with the guide nucleic acid component. The experiments done by the authors are well designed, well analyzed and clearly described, and the manuscript itself is very well written and will be of interest to a wide audience. The authors have succeeded in making excellent progress towards a molecular understanding of this complicated system, and the work comprises a significant advance in this interesting and difficult field. As such the work is certainly appropriate for publication in Nature Communications in largely it's present form, and the Supplementary Information sections are all also very useful and make admirably clear how each portion of the work was performed and analyzed.

Before the paper is accepted and published, however, I do want to point out one section of the study that is much less carefully and quantitatively defined than the rest of the work, and which – in my view – is weaker than the rest of the ms. This is the section at the end of the Results section where the authors discuss their work on the salt concentration dependence of the system and formulate their views on what they call a 'gliding' process for the translocation of the argonaute complex. In this part the authors discuss rather vague ideas such as that "Ago forcing little rearrangements in the ion cloud….", and comparably molecularly undefined statements, when actually – thanks to the work of Record, Manning, Oosawa and others -- our understanding of ion binding to DNA and comparable polyelectrolytes, and the effects of this binding on protein-nucleic acid complex formation and translocation of proteins such as transcription factors on DNA, is really quite well developed. Thus cation condensation onto the sugar-phosphate backbones of dsDNA (and in the present case ssDNA) -- is complete at cation concentrations of 2 to 3 mM, and neutralizes ~80% of the dsDNA phosphate charge (and ~70% of the phosphate charge on ssDNA, which has a lower charge density). It is the presence of these condensed ions that makes protein sliding on DNA possible, and the entropy of mixing of these cations with the solvent when they are displaced by the polyvalent cationic charges on the binding surface of the protein that provides the free energy that holds the protein on the DNA and permits the isoenergetic sliding reaction.

As a consequence – as clearly shown with model systems by Record and co-workers many years ago -- the binding constant for the protein component to the DNA polyelectrolyte decreases linearly in a log-log plot as a function of increasing counterion concentration. The balance of the binding free energy due to residual charge on the DNA phosphates decreases in a classical Debye-Huckel fashion with increasing cation concentration. Things are a bit more complicated when the dsDNA is also occupied by ssDNA secondary structure or other DNA-binding proteins (which tend to bind to the DNA via a positively charged binding cleft on the inner (facing the DNA) surface, while the 'backs' of these proteins (facing out into solution) tend to be net negatively charged and thus form a more 'messy' portion of the general polyelectrolyte surface of the overall protein-DNA complex, permitting the argonaute protein component to slide (or 'glide') over them in a one-dimensional random walk in much the same fashion as it slides over the DNA. A general description of these sliding ('gliding') models is presented in refs. 15 and 16 of the present Cui et al manuscript, and in the references to the work of the Record group contained in those papers. (See some key references below.*)

*Some key references on the control of protein-nucleic acid interactions and translocation by ion condensation:

Record et al., J. Mol. Biol., 107, 145-58, 1976.
Bleam et al, PNAS, 77, 3085-89,1980.
Winter et al. Biochemistry, 20, 20, 6961-77, 1981.

Of course, given the high quality of the rest of the ms, I think that the authors should have the option to decide whether they want to think about their 'gliding' experiments in this way, but it seems to me–

in contrast to the rest of their manuscript – that their present descriptions of the ‘gliding’ process and their vague references to “rearrangements of ion clouds”, etc., are unnecessarily undefined and vague in this otherwise taut and molecularly convincing ms.

In conclusion, however, I would reiterate that overall this is a fine manuscript that is certainly worthy of publication in Nature Communications, and that the above discussion of their ‘gliding’ model is intended for the authors’ consideration and not, in my mind, as a required change.

Review by Peter H. von Hippel

We would like to thank the reviewers for giving constructive feedback, which has helped us substantially improve the manuscript. We have made major revision of the manuscript and have conducted several sets of new experiments to consolidate our model. The main changes and the new experiments are:

- *Definitions (gliding vs sliding, sliding vs hopping, intersegmental transfer vs jumping)*

  Based on the comments from Reviewer 4, we dropped the term gliding and opted for the term sliding. Furthermore, we have included Supplementary Figures 7 and 8 to clarify the definitions (Reviewers 2, 4).

- *Clarification of theoretical modeling*

  Based on the comments from Reviewers 2 and 3, we have elaborated the definitions and interpretation of our kinetic model.

- *Extended form of blockade (dsDNA)*

  To further investigate the mechanism of intersegmental jumping, we have made constructs that contain a dsDNA blockade of different lengths and have observed that an increase in blockade length leads to increased dissociation. This suggests that Ago uses the flexibility of ssDNA for intersegmental jumping, answering questions from Reviewers #1, #2, and #3.

Below are point-by-point responses to the referees' points, with their points in bold and our response in normal face. In the main text we have highlighted major changes in yellow.

--

Response to reviewer #1:

This is a solid and well-performed study with novel insights and concepts on Ago protein-substrate interactions. It is a continuation of previous work of the same authors that presents validation of existing data but also novel findings. However, there are a number of issues that need to be clarified.

We'd like to thank the reviewer for the critical reading and the kind words.

1. One of the major issues with the study is that a very weak target site has been chosen. Three nt complementary would not be sufficient to bind Ago proteins to target sites. Such 'targets' might be very frequent in any genome and Ago proteins would dwell for a very short time. Although interesting insights into general kinetics on such sites are revealed, it remains unclear where this is similarly relevant for targets with higher degree of complementarity.

We thank the reviewer for raising this critical point. We want to emphasize that the use of "targets" here refers to the sequences designed to trap the Argonaute, as opposed to poly-T sites on the construct that share no sequence complementarity with the guide. Physiologically speaking, our "targets" indeed represent off-targets encountered by Ago, and not the cognate 'on-target' site. Hence, our assay is designed to detect Ago searching along a substrate with off-targets' – as would be the case in vivo.
As our assay in principle allows us to determine the escape time for any degree of complementarity, it

could in future studies be used to determine e.g. diffusion constants on more physiologically relevant RNA/DNA as well. We have now commented on this in Discussion as follows.

“Though we have shown how to determine the escape time for a 3-nt complimentary target, one could easily extend this study to determine the escape time for any complementarity, and so determine the diffusion constant on DNA with any base composition [Zwanzig, PNAS 1988]”

2. For the distal sites, one would assume that CbAgo would not move towards the target site but it would rather pass by due to DNA flexibility. One could probably test this more directly. The target sites should be close enough to Lin28 in order to prevent that the sites can reach each other. In this case, only one single FRET signal should be observe. This would further strengthen the model.

We thank the reviewer for proposing this idea, and we have accordingly tried moving the traps closer to prevent Ago from jumping past the Lin28. However, the proximity of the Lin28 protein next to the target site gave rise to highly varying FRET signals, presumably due to protein-protein interactions (see Figure R1). These technical difficulties make it hard to distinguish clearly the anticipated result from the nature of the protein-protein interaction.

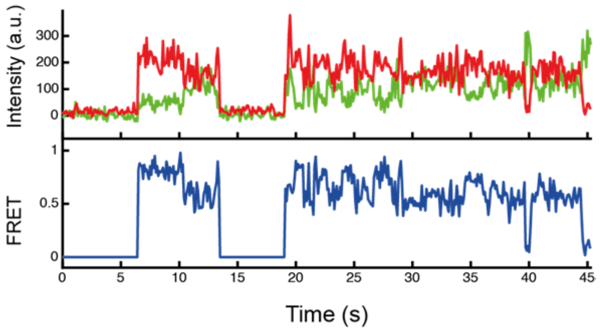

Figure R1. Example shuttling trace of Ago with Lin28 block with shortened flanks

While the new data did not meet the expectations of the reviewer, we also tried to probe the same issue using double stranded DNA of varying length to reduce the contact between the two traps. The results are in accordance with what would be expected for intersegmental jumping. We have now included an extra section discussing these experiments in the main text.

3. What is a '3 nucleotide (nt) subseed motif...'. It is clear what it is, but why such a cryptic explanation?

The subseed motif here refers to the finding that hAgo2 uses nucleotide 2-4 of the guide for initial target scanning [Chandradoss et al, Cell 2015]. We concede that the term may be confusing here and have decided to remove any mention of it. This should improve the clarity of the manuscript.

The remaining substrate is poly(T). Is there any particular reason why Ts have been selected? Could this also affect the outcome of the measurements?

We used a poly(N) sequence instead of a “natural” sequence to make sure that no partial trapping sequences are present near traps. T’s, known to make a most flexible structure, have been widely used for studying the polymer nature of single-stranded DNA (Murphy et al, Biophysical Journal 2004, https://www.ncbi.nlm.nih.gov/pubmed/15041689). We have considered using other nucleotides, but G’s and A’s were discarded since they are known to base-stack, resulting in stiffer constructs than one would find. C’s were discarded as C’s are part of the target sequence.

4. References to Figure 1 in the results section might be incorrect. Isn't Figure 1d (bottom of page 5) actually 1f? 1e and 1h are not mentioned at all.

We thank the reviewer for pointing this out, and have made the accompanying changes in the manuscript, and made sure all figures are referred to in the text.

5. On page 6, the authors state that the two target sites are separated by 22 nt. Figure 2A shows a linker of 19 nts.

We thank the reviewer for raising the issue. The 22 nt refers to the actual number of nucleotides that the Ago has to travel in order to reach the other target. While the spacing between the end of one target and the beginning of the other is indeed 19 nucleotides, the distance between the first nucleotide of each target is 19nt + 3nt (subseed length) = 22 nt. We have indicated the 22-nt distance in Figure 2a.

Along the same lines, on page 8 they state that they chose neighboring targets similar to the target separation in the previous assays (11nt). Where do the 11 nt come from?

The 11nt comes from Figure 3, where the first few data points are taken from the tandem target assay, with distances 11nt, 15nt, 18nt and 22nt. Since the 11nt is found well within the linear regime in the shuttling-time vs distance plot, we continued to use that distance when further probing the 3 target assay. The rationale is now made clearer in the text as follows.

"Next, we placed a third target on the tandem construct (**Figure 4a**), keeping the distance between each set of neighboring targets well within the regime for which we find good agreement to **Equation 1** using the assay discussed above (i.e. at 11 nt trap separation, see **Figure 3**)."

6. Reference to Hegge, Swarts et al. 2019 is incomplete.

That paper was published during this review process and we have made the accompanying changes to the reference.

Reviewer #2

In this paper, Cui et al. use a combination of single-molecule FRET experiments and theoretical modeling to interrogate the mechanism by which Argonaute proteins search for targets. They found that the Argonaute from C. butyricum (CbAgo) uses a new mode of lateral diffusion, which they termed “gliding”, for short-range target searching. For long-range search, CbAgo employs intersegmental jumps to reach distant sites. This combination of target-scanning modes allows Ago to bypass obstacles without hindrance. In general, the experiments are cleverly designed, especially the usage of multiple adjacent target sites to derive the escape rate. The data and analyses are both of high quality. The novel mechanism discovered here may be broadly applicable to other nucleic acid-guided proteins.

We thank the reviewer for the kind words and are happy that the reviewer appreciates the quality of the work and its broader applicability.

However, the manuscript is somewhat sloppily written. The authors should take more care with the text, figures, and references in terms of accuracy and consistency. At times key data are missing without explanation. The following issues need to be addressed before the paper can be accepted for publication at Nature Communications.

We have corrected all the errors that were pointed out to us.

1. The important distinction between “sliding” and “gliding” relies on the observation that CbAgo can bypass physical barriers. However, it is not entirely clear whether the Y-fork construct in Figure 5a really presents a barrier to CbAgo, since the ssDNA is essentially contiguous in this construct.

We thank the reviewer for the constructive criticism. As suggested by Reviewer 4, we have now opted to not use the term of gliding. In the revised manuscript, we instead stress that the sliding we have observed does not require tightly association with the backbone and thus can bypass junctions such as the discontinuous single-stranded structure of the Y-fork construct.

The protein roadblock in Figure 5c more likely presents a barrier, but that also depends on how the two single strands come out of Lin28b. A much more effective construct is the one depicted in Supplementary Figure 5, which contains a stretch of dsDNA that disrupts the continuous ssDNA. The authors seem to have done this experiment in Supplementary Fig. 5d-h. But these data were never discussed. Was CbAgo still able to shuttle between the two sites with a dsDNA roadblock in between? In fact, Supplementary Figure 5 contains many results, but was only briefly mentioned once in the text (Line 203-205).

We thank the reviewer for this suggestion. We have carried out new experiments and have found that Ago is able to shuttle between two sites separated by dsDNA if there is a long enough flank for them to come close to each other. For example, when 36 nt-separated targets contains a 17 bp blockade, Ago is still able to make it across. However, increasing the blockade size to 25 bp diminishes the probability for Ago to shuttle across to the other target. We have included an extra section in the main text (highlighted in yellow) treating the double stranded DNA blockade (see also our response to reviewer #1 – major comment 2).

2. The distinction between "gliding" and "hopping" relies on the "mild" versus "strong" dependence of the shuttling time on the ionic strength. What is the criterion used to define "mild" or "strong"? Is there a theoretical basis for this?

To our best knowledge, there is no theoretical prediction of what the *absolute* effect of salt should be on either diffusive mode, apart from that increasing the ionic strength will in general shift the equilibrium towards the (partially) unbound state during jumping. However, we do expect a difference in the *relative* effect of salt on sliding and hopping, as hopping includes large net displacements of ions, while sliding is not. In our results, 2-fold effect was observed for the short-range diffusion whereas 13-fold effect was observed for the long-range. As the latter mode, intersegmental transfer, is speculated to have similar salt-dependence as hopping, we concluded that the 2-fold effect is the signature of sliding, not hopping. We have elaborated this point in the revised manuscript:

"Based on the dependence of the single-target off-rate on the ionic strength (**Supplementary Fig. 1f**), we expect the rate of the intersegmental jumps to also be dependent on salt concentration, while sliding should only be moderately effected since it has no net effect on the ion condensation along the substrate"

3. The conclusion that CbAgo uses intersegmental jumps for target search across large distances mainly comes from the breakdown of the linear relationship between shuttling time and target separation at large distances shown in Figure 3. As the authors mentioned, such a mechanism relies on the ability of a distant segment of ssDNA to loop back into the proximity of CbAgo. It follows that the search efficiency should critically depend on the conformational entropy of the substrate, and should diminish on a force-stretched substrate (as demonstrated by Forget and Kowalczykowski, Nature 482:423-427 (2012)). Can the authors fix both ends of the ssDNA on surface and check whether CbAgo can still shuttle between two sites with a large separation? While such experiments are not required, the authors should at least discuss this possibility.

We would like to thank the reviewer for this suggestion. We had considered this approach ourselves, but a flow stretch assay would not give the required force to stretch a ssDNA, as significant forces (>6 pN) are required to overcome the entropic barrier. Alternative approaches are to attach a bead to the end of ssDNA and then apply for a flow, or fix both ends of ssDNA (as suggested). As we do not currently have either technique at our disposal, making it function in our group will delay the publication by an unforeseen period. Thereby we have instead included a mention of this possibility in Discussion as follows.

"Experimentally, one could further investigate the nature of intersegmental jumps through a combined tweezer-fluorescence single-molecule assay, where forces strong enough to pull on entropically coiled ssDNA can be applied"

Furthermore, the section on dsDNA blockade that we have now included probes the same effect by progressively separating the targets in space, not by force but by making the intervening substrate stiffer; this has the added benefit precluding sliding between targets, making the interpretation of the results easier.

4. The definition of "shuttling time" is confusing. I initially thought it represents the transition time between different FRET states. Then I realized that it actually represents the dwell time of each FRET state. Please specifically clarify this term or use another term.

It indeed refers to the ‘apparent’ dwell time of each FRET state. However, as one of our major points is that due to our limited time resolution we cannot fully resolve the many escapes and recapture events actually present between shuttling events, we did not want to refer to it as the ‘actual’ dwell time of the FRET state. We now explicitly define the shuttling time as the time between shuttling events as follows.

“The time needed for FRET transitions to occur (named “shuttling time”) is equivalent to the apparent dwell time at a single FRET state.”

5. Instead of a supplementary table, a cartoon figure illustrating the different target search mechanisms (sliding, hopping, gliding, intersegmental jumping, etc.) and the combined searching mode of gliding and intersegmental jumping employed by CbAgo would be more informative. And I don’t understand the difference between intersegmental jumping and intersegmental transfer.

We have included a cartoon figure (Supplementary Figure 7) for clarification. Please note that in our interpretation of the literature, intersegmental ‘transfer’ implies that a protein is transferred between two segments by utilizing ‘two’ binding sites, and never loses contact with the substrate. In this work we used the term of intersegmental ‘jumping’ following (Broek *et. al* 2008) to refer to transfers not relying on two binding sites (no constant contact).

Minor points:

1. In Line 120-125, a single-exponential distribution is also consistent with stable binding to the target without any transient escape, whose lifetime would be governed by a single dissociation rate. How could the authors distinguish between the two scenarios? Is it because of the observed decrease in dwell time from 37 s to 1.7 s when changing from one target to two targets? Please comment.

We are sorry that this was not clear in the main text, but the logic runs in the reverse direction. Naively, one might have thought that we could rule out transient escapes by considering the distribution of apparent escape times. We then show theoretically that this is not so, and proceed to other ways of distinguishing the scenarios (e.g. the dependence on trap separation etc., as well as the change in dwell time when introducing a second target, as the reviewer points out). We have reworked the relevant text to make this clearer.

2. What are the dye positions for dual-target constructs with large distances (e.g., the 120-nt substrate in Fig. 3)? Can binding events to both sites still be detected and distinguished by FRET?

In all constructs, the Cy5 fluorophore is kept close to one site, 19 nt away from the CTC motif (see **Supplementary table 2**, partially included here for reference) while increasing the distance between sites to the corresponding separation.

8nt double target 120nt separation 171mer	5' –TTT TTT TTT TTT TTA CTA CCT CTT TTT TTT TTT TTT TTT TTT TTT TTT TTT TTT TTT TTT TTT TTT TTT TTT TTT TTT TTT TTT TTT TTT TTT TTT TTT TTT TTT TTT TTT TTT TTT TTT TT/iAmMC6T/ TTT TTT TTT TTA CTA CCT CTT TTT TTT TTT TTT TTT TTT TTT TTT TTT TT/biotin dT//Phos/-3'

This design results in a clear distinguishable signature for all constructs. For the larger distances it becomes easier to distinguish the FRET levels as the difference between two states become more prominent (see Figures R2 and R3). We have also included example traces for other tandem constructs in Supplemental Figure 9.

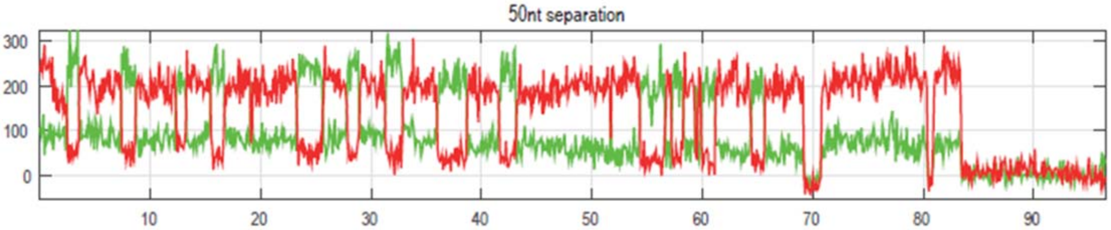

Figure R2 A representative trace containing a shuttling signature for N=3 for CbAgo on a construct with 50 nt separation between CTC motifs

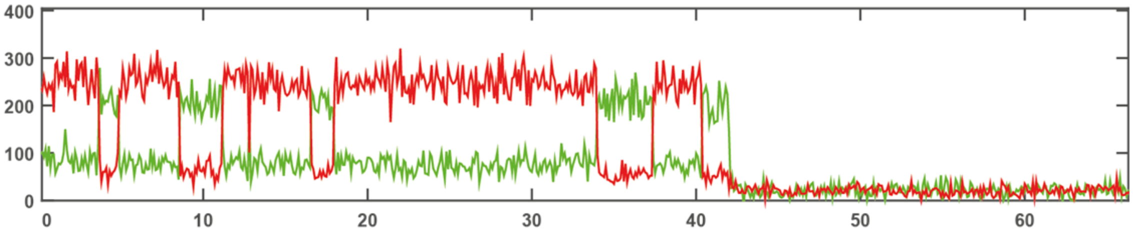

Figure R1. A representative trace containing a shuttling signature for N=3 for CbAgo on a construct with 120 nt separation between CTC motifs

3. In Figure 4d, the "shuttling times" for A-to-B and B-to-A events should be included.

The shuttling times A-to-B have been included as per request in Figure 4d. The shuttling times B-to-A are actually also included in the middle bar plot, since what is actually shown in Figure 4d are the dwell times per state, so the dwell times in B contain both the times B-to-A and B-to-C. We have therefore made the accompanying changes in the figure and the main text to indicate that.

4. The shuttling time for 11-nt separation reported in Figure 3 and Figure 5b is not consistent.

The experimental results are from different sets of data acquired in different days. While the exact numbers differ, the numbers are still within the error margin, but all mentions have been edited for consistency.

5. Instead of a categorically distinct substrate, the "no target" substrate in Figure 1c is actually a continuation of the series of substrates tested in Supplementary Figure 1c, with a guide length of N=1. The "T" in the poly(T) target sequence complements the "A" at nt #2 of the guide. The results for this substrate should be incorporate into Supplementary Fig. 1c for direct comparison. By the same token, a substrate with a guide length of N=2 would be nice to test too. Perhaps the authors already have the results?

We have included the results for varying complementarity (N = 1, 2, …) in the supplementary file (Supplementary Fig 1c). We have also revised the main text, replacing “no target” with “N = 1”.

6. The figures are not arranged in the order in which they are referenced. In some cases the wrong figure is referred to. For example, in Line 187, “The exponential distribution of the dwell times (Supplementary Fig. 4c) suggests that…” should be Supplementary Fig. 4b. In Line 263, “…while our Y-fork experiments show that CbAgo need not be in constant physical contact with its substrate during this process (Figure 4b).” Does Fig. 4b tell us this?

We are sorry for the errors in figures and have made the accompanying changes.

7. There are numerous typos throughout the manuscript (e.g., line 161, line 275, line 322).

We are sorry for the errors of this and others in the manuscript and have corrected all we have been able to find.

Reviewer #3

In "Argonaute bypasses cellular obstacles without hindrance during target search", Cui et al. present single molecule FRET (smFRET) experiments to elucidate the ssDNA target search mechanism of an Argonaute protein from the bacterium Clostridium butyricum (CbAgo). Through a series of single-molecule measurements on ssDNA with 1 to 3 target sequences, the authors determine that Ago identifies targets through facilitated diffusion, consisting of periods of lateral 1D diffusion over short distances with periods of intersegmental transfer, allowing Ago to bypass barriers such as DNA-bound proteins.

Overall this is a well-designed study on an important class of protein. The experiments are cleverly designed and the data convincing. However, there are important criticisms the authors need to address, as detailed below. The most important point is that the novelty of the results is unclear. Facilitated diffusion is a well-established mechanism of target search shown for a number of DNA binding proteins (transcription factors, restriction enzymes, etc.), and I am not convinced that the difference in dsDNA vs. ssDNA target search is that significant. I believe the authors overstate this point. While the quantitative model proposed is useful, the results themselves are unsurprising. Thus, it is unclear that the work rises to the level expected for publication in Nature Communications. The authors should address this point (and others enumerated below) before a recommendation on the manuscript can be made.

We appreciate the constructive feedback of the reviewer, and the chance to make our case more clearly. However, we respectfully disagree with the statement that the results are unsurprising.

This study uses the high spatiotemporal resolution of FRET to observe that nucleic acid-guided proteins undergo lateral diffusion instead of relying on guide-target base-pairing interactions alone, which was not known from the literature. Target search on dsDNA has indeed been better-characterized, but these substrates are stiff and rarely form secondary structures, making the results hard to generalize to our system. Therefore, the finding that Ago uses search strategies that are uninhibited by secondary structures and allows the protein to bypass roadblocks even at relatively short distances (30 nt) is of great value to understanding these important search processes.

In addition, the assay and analysis we introduce could in principle be used to experimentally measure and predict the effective diffusion coefficient as a function of sequence, with a resolution that is not accessible through other single-molecule method studies (DNA curtains, flow stretch etc). This in turn may allow us to better understand how the genomic sequence context would impact targeting for nucleic-acid guided proteins.

Specific points:

1. In their model, the shuttling time is proportional to the distance between target sites (Eq. (1)), which is corroborated by the data (Fig. 3). This seems counterintuitive, since a diffusive mechanism should lead to $x^2 \sim t$. Could the authors provide a simple explanation for this? Is this because the rate-limiting step is not the 1D diffusion itself but release of Ago from the binding site? This is not clearly explained in the text or SI.

As the reviewer correctly stated, the linear dependence is indeed due to the change in probability of diffusing across the construct without getting retrapped, not from the 1D diffusion itself. Derivation of

this first-passage time is shown in Supplementary Information. We have now included sentences below **Equation 1** to this effect as follows:

"The linear dependence of the shuttling time with trap separation might seem puzzling at first, given that diffusive timescales usually show a quadratic dependence on distances. Here though, it is not the diffusive steps themselves that directly contributes to the shuttling time, but rather the changing probability to getting retrapped before shuttling."

2. As pointed out in the text (lines 227-228), for intersegmental transfer to occur, ssDNA must loop back into proximity of the bound Ago. The authors should be able to expand their model by quantitatively modeling the kinetics of intersegmental transfer. This can be done by accounting for the probability of looping, models for which can be found in the literature.

We thank the reviewer for this suggestion. We are performing further in-depth theoretical studies on the effect jumps have on both the measured shuttling events and the search time on a genome. As this requires intensive theoretical work and goes beyond the scope of this manuscript, we aim to publish it as a separate work.

3. Looping is highly dependent on DNA flexibility, and the authors could explicitly test their by introducing stiff dsDNA sections between target sites.

We thank the reviewer for this highly valuable suggestion. As stated before in response to Reviewers #1 and #2, we have included an extra section (see section "CbAgo relies on flexibility of DNA segments for bypassing blockades") where we explicitly test the effect of an extra dsDNA section in between targets. We noticed that a 17-bp long dsDNA block minimally inhibited shuttling. However, increasing the blockade further to 25 bp, we noticed a significant drop in the shuttling probability. When we disabled DNA coiling from one side by truncating the DNA beyond the target, this resulted in a ~50% drop in shuttling probability, indicating that DNA coiling is a significant factor for Ago to reach distant sites.

4. On a related point, the authors draw several conclusions from their measurements of search kinetics at different ionic strengths. However, DNA flexibility is also dependent on ionic strength. Is it possible that the changes in target search kinetics the authors observe upon increasing ionic strength are in fact due to changes in DNA flexibility, rather than changes in protein affinity to DNA (lines 243-249)? This alternative should be considered explicitly.

We would like to thank the reviewers for raising this point. We had considered this possibility, but several studies showed that the persistence length does not vary substantially (~20 Å to 14Å) within our measured range of salt conditions (10-200 mM NaCl and 1 mM $MnCl_2$). We have now made this explicitly in the text by citing a paper [Chen et al, PNAS, 2012])

5. Line 275-276: "CbAgo uses a target search mechanism distinct from other known DNA binding proteins." As stated above in this review, facilitated diffusion has been exhibited in a host of other DNA binding proteins during target search. Even if one constrains oneself to ssDNA binding proteins, single-stranded DNA binding proteins (SSB) from E. coli have been shown to exhibit both 1D diffusion and intersegmental transfer. Therefore, this is not a new finding.

We appreciate this criticism. We have toned down our claim in regards to DNA binding proteins. While we agree with the reviewer's criticism, we want to kindly emphasize the difference between the SSB protein and the Ago complex. Unlike Ago-mediated target search, where monovalent DNA-DNA interactions are required, SSB binding relies on multivalent and non-sequence specific protein-DNA interactions (Roy et al, Nature, 2009).

6. Line 276-277: "Since the CbAgo searches on ssDNA, not dsDNA, it cannot make use of the structural regularity of double stranded DNA." This argument is made to highlight the difference between diffusion on ssDNA and dsDNA, but I do not find it compelling. It is unclear what evidence exists demonstrating that the structural regularity of dsDNA is essential for 1D diffusion. The authors need to motivate this statement better.

We thank the reviewer for pointing this. Upon further consideration we agree and have now removed this statement.

7. Line 327-328: "dsDNA is substantially more conducive for tight interactions than flexible single stranded nucleic acids". I am not convinced this is true. Single-stranded DNA binding proteins (SSB) and recombinases RecA/Rad51 are examples of proteins that bind to ssDNA with high affinity. If anything, one could make the opposite argument, since the high flexibility of ssDNA would provide a low energy cost to deformation by protein binding, in contrast to dsDNA (e.g. exhibited by transcription factors, restriction enzymes).

We'd like to thank the reviewer for raising this fair criticism. Similar to the statement above of "structural regularity", we agree and have decided not to include this point in the discussion.

In addition there are several typographical and grammatical errors in the text that need to be corrected:

Main text:
Line 155: "we kinetically model how the this time depends on…" should have one of the two articles, "the" or "this", removed
Line 333: "orientate" should be "orient"
SI:
Section A, paragraph 1: "inital assocation" should be "initial association"
Section B, paragraph 2: "succesful" should be "successful"
Section B, paragraph 3: "accross" should be "across"
Section D, paragraph 2: "precedure" should be "procedure"

We are thankful to the reviewer for pointing out these errors. We have corrected them and others in the revised version.

Reviewer #4

This is a largely excellent manuscript that deals with an important subject -- namely, how -- in molecular detail -- do genome-regulatory proteins find their target sites on DNA? More specifically, and unlike the transcription factors that have largely been studied in this context in the past, the argonaute systems studied in this work operate by finding their targets on single-stranded (ss) DNA by use of a small nucleic acid guide component that is essential to final recognition of the target sequence. Thus these protein-nucleic acid complexes search the target by a multi-step process; first binding to the overall ssDNA target by non-sequence-specific electrostatic interactions with the protein component that permit various forms of 'lateral diffusion', and then recognizing the target by sequence complementarity with the nucleic acid guide component. The authors use a well-designed series of protein-nucleic acid labeled FRET constructs to study the rates and extents of translocation events in an attempt to define – at the molecular level -- how these argonaute complexes scan across and then recognize their ssDNA target sequences in the DNA genome.

The results obtained suggest that the overall search mechanism can be considered as the sum of two separate processes:

(i) an electrostatically-stabilized general binding of the protein component of the argonaute complex to the overall ssDNA target on which it undergoes 'lateral diffusion' in the course of 'scanning' for the actual target site; and

(ii) local recognition of the target sequence itself by complementary base-pairing with the guide nucleic acid component.

The experiments done by the authors are well designed, well analyzed and clearly described, and the manuscript itself is very well written and will be of interest to a wide audience. The authors have succeeded in making excellent progress towards a molecular understanding of this complicated system, and the work comprises a significant advance in this interesting and difficult field. As such the work is certainly appropriate for publication in Nature Communications in largely it's present form, and the Supplementary Information sections are all also very useful and make admirably clear how each portion of the work was performed and analyzed.

We are grateful to Prof. Hippel for the thorough reading of our manuscript, the appreciation of our work, and his kind words.

Before the paper is accepted and published, however, I do want to point out one section of the study that is much less carefully and quantitatively defined than the rest of the work, and which – in my view – is weaker than the rest of the ms. This is the section at the end of the Results section where the authors discuss their work on the salt concentration dependence of the system and formulate their views on what they call a 'gliding' process for the translocation of the argonaute complex. In this part the authors discuss rather vague ideas such as that "Ago forcing little rearrangements in the ion cloud….", and comparably molecularly undefined statements, when actually – thanks to the work of Record, Manning, Oosawa and others -- our understanding of ion binding to DNA and comparable polyelectrolytes, and the effects of this binding on protein-nucleic acid complex formation and translocation of proteins such as

transcription factors on DNA, is really quite well developed. Thus cation condensation onto the sugar-phosphate backbones of dsDNA (and in the present case ssDNA) -- is complete at cation concentrations of 2 to 3 mM, and neutralizes ~80% of the dsDNA phosphate charge (and ~70% of the phosphate charge on ssDNA, which has a lower charge density). It is the presence of these condensed ions that makes protein sliding on DNA possible, and the entropy of mixing of these cations with the solvent when they are displaced by the polyvalent cationic charges on the binding surface of the protein that provides the free energy that holds the protein on the DNA and permits the isoenergetic sliding reaction.

As a consequence – as clearly shown with model systems by Record and co-workers many years ago -- the binding constant for the protein component to the DNA polyelectrolyte decreases linearly in a log-log plot as a function of increasing counterion concentration. The balance of the binding free energy due to residual charge on the DNA phosphates decreases in a classical Debye-Huckel fashion with increasing cation concentration. Things are a bit more complicated when the dsDNA is also occupied by ssDNA secondary structure or other DNA-binding proteins (which tend to bind to the DNA via a positively charged binding cleft on the inner (facing the DNA) surface, while the 'backs' of these proteins (facing out into solution) tend to be net negatively charged and thus form a more 'messy' portion of the general polyelectrolyte surface of the overall protein-DNA complex, permitting the argonaute protein component to slide (or 'glide') over them in a one-dimensional random walk in much the same fashion as it slides over the DNA. A general description of these sliding ('gliding') models is presented in refs. 15 and 16 of the present Cui et al manuscript, and in the references to the work of the Record group contained in those papers. (See some key references below.*)

***Some key references on the control of protein-nucleic acid interactions and translocation by ion condensation:**

Record et al., J. Mol. Biol., 107, 145-58, 1976.
Bleam et al, PNAS, 77, 3085-89,1980.
Winter et al. Biochemistry, 20, 20, 6961-77, 1981.

We thank Prof Hippel for his thorough and expert exposition on the effects of counter ions, and we especially appreciate pointers to the relevant literature. Prof Hippel is also fair in his criticism that the last section of the results may be the least strong, as the phenomenon of sliding in relation to counter ion condensation is already well characterized. In light of this, we have decided to dwell less on various possible effects of counter ions, but retain only two central things: 1) that the equilibrium is shifted toward unbinding for increased counter ion concentrations, and 2) that we expect intersegmental jumping to have to perturb to counter ion cloud more than sliding, and so be more effected by concentration changes. We have also dropped our term gliding, and have instead opted for using sliding throughout, but emphasizing that it is loose enough to allow Ago to slide past secondary structures. We hope these changes have made the overall manuscript more convincing and of higher quality

Of course, given the high quality of the rest of the ms, I think that the authors should have the option to decide whether they want to think about their 'gliding' experiments in this way, but it seems to me– in contrast to the rest of their manuscript – that their present descriptions of the 'gliding' process and their vague references to "rearrangements of ion clouds", etc., are unnecessarily undefined and vague in this otherwise taut and molecularly convincing ms.

In conclusion, however, I would reiterate that overall this is a fine manuscript that is certainly worthy of publication in Nature Communications, and that the above discussion of their 'gliding' model is intended for the authors' consideration and not, in my mind, as a required change.

Review by Peter H. von Hippel

REVIEWERS' COMMENTS:

Reviewer #1 (Remarks to the Author):

The authors have addressed all points that I had raised on the previous version of their manuscript. They have performed additional experiments and explained some of the issues in more detail. The manuscript is now clearer and I am satisfied with the response to my review.

Reviewer #2 (Remarks to the Author):

The authors have addressed all of my concerns in the revised manuscript. I now recommend its publication at Nature Communications.

Reviewer #3 (Remarks to the Author):

In their revision to "Argonaute bypasses cellular obstacles without hindrance during target search", Cui et al. have addressed many of the criticisms and comments of the reviewers. In particular, the authors have collected additional smFRET data with stiffer constructs containing dsDNA "blockades", which lead to reduced Ago shuttling probability, consistent with their model that this behavior is mediated by DNA looping.

A main criticism in my previous review was over the novelty of the results with regards to the facilitated diffusion mechanism, which has been observed in many other systems. The authors make a compelling argument that their findings are important for Ago-like proteins. In particular, I agree with their statement that "the finding that Ago uses search strategies that are uninhibited by secondary structures and allows the protein to bypass roadblocks even at relatively short distances (30 nt) is of great value to understanding these important search processes". Also, the authors have largely toned down statements I felt overstated the generality of their findings to all DNA binding proteins. In sum, I believe the authors have addressed the main issues of the manuscript, and I think the work is suitable for publication in Nature Communications.

Specific comments:
The authors still need to be careful with some of the general statements they make, which could be misconstrued. For example, in line 272 the statement that "this mode of loose-contact sliding has not been reported for any nucleic-acid guided proteins" could easily be misinterpreted. One could argue that "loose-contact" sliding has been observed in other systems. I think it would be better to be clear here that they mean "the ability to bypass junctions and roadblocks".

Line 127: "…targets with complementary increased…" I think the authors mean "complementarity" here.
Line 252: "…translocation being dominate by sliding over short distances" I think the authors mean "dominant" here.
Line 322: "the absence of cooperative binding was recentley reported" should be "recently".

Throughout the main text, several Supplementary Figure panels are not referenced in the text (Suppl. Fig. 2a-e, 4a, c-g), and three Supplementary Figures are not referenced at all (Supp. Figs. 7, 8, and 9). This leaves the impression that these plots are nonessential, in which case they could be removed. The text should point to all figure panels important enough to include in the manuscript.

In Supplementary Figure 4a, it is unclear what the three FRET distributions (in different colors) represent. The caption does not clarify what is being plotted.

Reviewer #4 (Remarks to the Author):

Thank you for your revised manuscript and helpful covering letter detailing how you have responded to the various concerns raised by the reviewers. I believe that you have largely dealt successfully with the comments that I raised (reviewer #4), and also -- to the extent that I can judge -- with the comments raised by the other reviewers.

First, I am pleased that you have agreed to use the term "sliding" instead of "gliding" for the facilitated diffusion process of the argonaute complex along the contour of the nucleic acid substrate, since the motion that you are referring to is just a generalized extension of the 'sliding' mechanism as the term has been used for many years. I think that the supplementary Figure 7 that you use to illustrate these various translocation mechanisms is helpful. I suggest that to simplify things still further that you might also consider that the "intersegment transfer" process can by generalized as well, since the two binding sites used in this process need not be identical -- and in fact non-specific binding can serve as a second binding site with significant probability of transfer as long as the binding affinities of the two sites are not too different -- which is likely the case for the weak 'specific' binding interactions involved here.

This also makes the use of the "intesegmental jump" term unnecessary, since this merely becomes a generalized form of "hopping", which is a general term for the protein complex moving about on the nucleic acid substrate, and differs from classical 3-D diffusion only in the fact that the complex is more likely to return to a position near the nucleic acid site from which it was released, rather than being fully released into the solution. And of course a nearby site would include those transiently close by because of looping, especially for single-stranded nucleic acid substrates, rather than simply those that are nearby because of proximity along the substrate contour. This generalized form of hopping would likely also show the differences in condensed ion displacement that you illustrate in Figure 7 for the difference between 'sliding' and 'hopping' in the first two parts of your schematic in Figure 7. These changes would simplify things and also fit in well with cartoon representation in Figure 8, as well as the way that the "intersegmental transfer" and "hopping" terms have been used for these translocation processes when dealing with intervening proteins (polymerases, nucleosomes, etc.) in the literature on facilitated diffusion on dsDNA.

An additional minor point: in reading through the ms and the SI sections I noted that the word 'gliding' has not been replaced by 'sliding' in several places in the SI section entitled "Derivations: Argonaute bypasses...", including the title of Section B. These changes should be made.

Otherwise I am happy now to recommend this revised version of your ms for publication. I believe that it comprises a significant addition to the literature of this field and its generalization to complexes involving "guide RNAs" and transfer along ssDNA, such as those described here. Congratulations on a very nice piece of work!

Reviewed by Peter H. von Hippel

REVIEWERS' COMMENTS:

Reviewer #1 (Remarks to the Author):

The authors have addressed all points that I had raised on the previous version of their manuscript. They have performed additional experiments and explained some of the issues in more detail. The manuscript is now clearer and I am satisfied with the response to my review.

We thank the reviewers for going through our responses and for their final decision.

Reviewer #2 (Remarks to the Author):

The authors have addressed all of my concerns in the revised manuscript. I now recommend its publication at Nature Communications.

We thank the reviewers for going through our responses and for their final decision.

Reviewer #3 (Remarks to the Author):

In their revision to "Argonaute bypasses cellular obstacles without hindrance during target search", Cui et al. have addressed many of the criticisms and comments of the reviewers. In particular, the authors have collected additional smFRET data with stiffer constructs containing dsDNA "blockades", which lead to reduced Ago shuttling probability, consistent with their model that this behavior is mediated by DNA looping.

A main criticism in my previous review was over the novelty of the results with regards to the facilitated diffusion mechanism, which has been observed in many other systems. The authors make a compelling argument that their findings are important for Ago-like proteins. In particular, I agree with their statement that "the finding that Ago uses search strategies that are uninhibited by secondary structures and allows the protein to bypass roadblocks even at relatively short distances (30 nt) is of great value to understanding these important search processes". Also, the authors have largely toned down statements I felt overstated the generality of their findings to all DNA binding proteins. In sum, I believe the authors have addressed the main issues of the manuscript, and I think the work is suitable for publication in Nature Communications.

We thank the reviewer for re-evaluating the importance of our findings and supporting the publication.

Specific comments:
The authors still need to be careful with some of the general statements they make, which could be misconstrued. For example, in line 272 the statement that "this mode of loose-contact sliding has not been reported for any nucleic-acid guided proteins" could easily be misinterpreted. One could argue that "loose-contact" sliding has been observed in other systems. I think it would be

better to be clear here that they mean “the ability to bypass junctions and roadblocks”.

We thank the reviewer for carefully reading the manuscript and have rephrased the sentence.

Line 127: “…targets with complementary increased…” I think the authors mean “complementarity” here.

We have corrected the mistake.

Line 252: “…translocation being dominate by sliding over short distances” I think the authors mean “dominant” here.

We have corrected the mistake.

Line 322: “the absence of cooperative binding was recentley reported” should be “recently”.

We have corrected the mistake.

Throughout the main text, several Supplementary Figure panels are not referenced in the text (Suppl. Fig. 2a-e, 4a, c-g), and three Supplementary Figures are not referenced at all (Supp. Figs. 7, 8, and 9). This leaves the impression that these plots are nonessential, in which case they could be removed. The text should point to all figure panels important enough to include in the manuscript.

We made references to the respective panels and respective Figures in the main text. Also Supplementary Figure 7 was shuffled to a more logical order to accommodate the main text.

In Supplementary Figure 4a, it is unclear what the three FRET distributions (in different colors) represent. The caption does not clarify what is being plotted.
We thank the reviewer for pointing this out and added a legend to the supplementary figure for clarity.

Reviewer #4 (Remarks to the Author):

Thank you for your revised manuscript and helpful covering letter detailing how you have responded to the various concerns raised by the reviewers. I believe that you have largely dealt successfully with the comments that I raised (reviewer #4), and also -- to the extent that I can judge -- with the comments raised by the other reviewers.

First, I am pleased that you have agreed to use the term "sliding" instead of "gliding" for the facilitated diffusion process of the argonaute complex along the contour of the nucleic acid

substrate, since the motion that you are referring to is just a generalized extension of the 'sliding' mechanism as the term has been used for many years. I think that the supplementary Figure 7 that you use to illustrate these various translocation mechanisms is helpful. I suggest that to simplify things still further that you might also consider that the "intersegment transfer" process can by generalized as well, since the two binding sites used in this process need not be identical -- and in fact non-specific binding can serve as a second binding site with significant probability of transfer as long as the binding affinities of the two sites are not too different -- which is likely the case for the weak 'specific' binding interactions involved here.

This also makes the use of the "intesegmental jump" term unnecessary, since this merely becomes a generalized form of "hopping", which is a general term for the protein complex moving about on the nucleic acid substrate, and differs from classical 3-D diffusion only in the fact that the complex is more likely to return to a position near the nucleic acid site from which it was released, rather than being fully released into the solution. And of course a nearby site would include those transiently close by because of looping, especially for single-stranded nucleic acid substrates, rather than simply those that are nearby because of proximity along the substrate contour. This generalized form of hopping would likely also show the differences in condensed ion displacement that you illustrate in Figure 7 for the difference between 'sliding' and 'hopping' in the first two parts of your schematic in Figure 7. These changes would simplify things and also fit in well with cartoon representation in Figure 8, as well as the way that the "intersegmental transfer" and "hopping" terms have been used for these translocation processes when dealing with intervening proteins (polymerases, nucleosomes, etc.) in the literature on facilitated diffusion on dsDNA.

We recognize the confusion regarding the use of the terms within the biophysics community and agree largely with the points made here. We have avoided the use of intersegmental jumping. However, to remain consistent with the literature as it is currently, instead of not using the term of intersegmental transfer, we have added a sentence in the main text clarifying that the hops to neighbouring segments are named intersegmental transfer. As such, we have changed supplementary figure 8 to further clarify this issue.

An additional minor point: in reading through the ms and the SI sections I noted that the word 'gliding' has not been replaced by 'sliding' in several places in the SI section entitled "Derivations: Argonaute bypasses...", including the title of Section B. These changes should be made.

We have addressed these mistakes in the SI section as well.

Otherwise I am happy now to recommend this revised version of your ms for publication. I believe that it comprises a significant addition to the literature of this field and its generalization to complexes involving "guide RNAs" and transfer along ssDNA, such as those described here. Congratulations on a very nice piece of work!

We are very grateful for the extremely kind words and appreciate the helpful comments throughout the review process to improve the quality of the work in general.

Reviewed by Peter H. von Hippel
